# Single-molecule localization microscopy reveals molecular transactions during RAD51 filament assembly at cellular DNA damage sites

**DOI:** 10.1093/nar/gkx1303

**Published:** 2018-01-04

**Authors:** Kalina T Haas, MiYoung Lee, Alessandro Esposito, Ashok R Venkitaraman

**Affiliations:** The Medical Research Council Cancer Unit, University of Cambridge, Hills Road, Cambridge CB2 0XZ, UK

## Abstract

RAD51 recombinase assembles on single-stranded (ss)DNA substrates exposed by DNA end-resection to initiate homologous recombination (HR), a process fundamental to genome integrity. RAD51 assembly has been characterized using purified proteins, but its ultrastructural topography in the cell nucleus is unexplored. Here, we combine cell genetics with single-molecule localization microscopy and a palette of bespoke analytical tools, to visualize molecular transactions during RAD51 assembly in the cellular milieu at resolutions approaching 30–40 nm. In several human cell types, RAD51 focalizes in clusters that progressively extend into long filaments, which abut—but do not overlap—with globular bundles of replication protein A (RPA). Extended filaments alter topographically over time, suggestive of succeeding steps in HR. In cells depleted of the tumor suppressor protein BRCA2, or overexpressing its RAD51-binding BRC repeats, RAD51 fails to assemble at damage sites, although RPA accumulates unhindered. By contrast, in cells lacking a BRCA2 carboxyl (C)-terminal region targeted by cancer-causing mutations, damage-induced RAD51 assemblies initiate but do not extend into filaments. We suggest a model wherein RAD51 assembly proceeds concurrently with end-resection at adjacent sites, via an initiation step dependent on the BRC repeats, followed by filament extension through the C-terminal region of BRCA2.

## INTRODUCTION

HR preserves genomic integrity through its role in DNA double-strand break (DSB) repair (reviewed in (1,2)). Numerous cytological and biochemical studies in yeast, bacterial and mammalian systems have pioneered current understanding of the steps leading to HR (reviewed in (3–6)). Two key events—the resection of DNA ends and the assembly of RAD51 recombinase on the exposed DNA substrates—initiate HR at DSBs. In human somatic cells, DSB ends are processed by the MRE11–RAD50–Nibrin (MRN) complex, and end-resected by nucleases including BLM-DNA2 or EXO1, to expose ssDNA tracts (7–11). Biochemical (12–16) and cytological studies carried out in meiotic (17) or mitotic (18) yeast cells suggest that these tracts are rapidly bound by Replication protein A (RPA), an abundant ssDNA-binding protein, which regulates the activity of end-resecting nucleases, and protects ssDNA (11,19,20). Resected ssDNA establishes a substrate upon which RAD51 recombinase assembles into ordered nucleoprotein filaments, which catalyze homologous pairing and strand exchange with homologous dsDNA to execute HR (21). Human RAD51 assembly has been characterized *in vitro* with biochemical and structural studies using purified proteins (22–26), which suggest that the tumor suppressor protein, BRCA2, is an essential mediator that targets RAD51 to appropriate DNA substrates to correctly order the biochemical reactions leading to HR (27–32).

Diffraction-limited microscopy shows that RAD51 forms nuclear foci at sites of DNA damage associated with RPA accumulation (19), and that RAD51 foci formation is impaired in BRCA2-deficient cells (33). A pioneering study using super-resolution microscopy to examine Rad51 foci in yeast cells (which lack a BRCA2 homologue) reveals either small clusters separated by <200 nm in meiotic cells, or filaments ∼70 nm wide and >1000 nm long formed after Rad51 overexpression in mitotic cells (34). The distribution of RAD51, BRCA2 and RPA in damage-induced foci formed in human cells has also been studied by super-resolution methods (53). However, the ultrastructural topography of DNA end-resection and RAD51 filament assembly at somatic DSBs in human cells, and its regulation by BRCA2, remains unexplored.

A particular challenge to the characterization of molecular events at cellular DSB sites is the complexity of underlying biochemical steps suggested by *in vitro* studies. For instance, RPA inhibits RAD51 filament formation on ssDNA *in vitro* by competing for the same binding sites, but also enables productive RAD51 binding by removing ssDNA secondary structures (12,35,36). RPA binds to RAD51, but this interaction is insufficient to displace RPA from ssDNA (37). BRCA2—which also binds to RPA through an N-terminal region—may help to overcome the kinetic barrier posed by RPA, by stimulating RPA dissociation from ssDNA *in vitro* and promoting RAD51 filament formation in its place (31,38,39). Moreover, human BRCA2 can bind at least six monomers of RAD51 (30), via an array of eight evolutionarily conserved motifs (BRC repeats), each of ∼35 residues (40–42). *In vitro* biochemical studies suggest that certain BRC repeats, typified by human BRC4, bind to monomeric RAD51 with relatively high affinity (40). At high stoichiometry, BRC4 peptide can inhibit RAD51 filament assembly, and dissolve pre-formed RAD51 filaments (32,43). Indeed, crystallographic studies show that BRC4 mimics the RAD51 monomer–monomer interaction, preventing RAD51 oligomerization and thus, RAD51 foci formation, when overexpressed in cells (44,45). Remarkably, however, sub-stoichiometric concentrations of BRC4 peptide can redirect RAD51 from dsDNA to ssDNA, and inhibit ssDNA-stimulated RAD51 ATPase activity, thereby stabilizing RAD51 filaments on ssDNA (27,28,46). Additionally, biochemical evidence also suggests that certain other BRC repeats, typified by human BRC5–8, may bind to RAD51 oligomers rather than monomers, promoting filament assembly (47). A distinct carboxyl (C)-terminal domain of BRCA2, which is distinct in sequence from the BRC repeats, also stabilizes RAD51 filaments and counteracts *in vitro* the ability of BRC1–4 to dissociate RAD51 from DNA (48,49). How these biochemical features of RAD51 assembly *in vitro* are reflected in the molecular intermediates detected at cellular somatic DSB sites remains unclear. In living cells, multimeric BRCA2 co-diffuses with RAD51 in large complexes that undergo dynamic changes after DNA damage (50,51). Scanning force microscopy combined with live-cell imaging and super-resolution optical microscopy revealed large, extended multimeric complexes of BRCA2-RAD51 up to several hundred nm in size (52), which may promote the delivery of multiple RAD51 oligomers for assembly at damage sites. Thus, it has been challenging to map the choreography of molecular transactions during DNA end-resection and RAD51 assembly from the biochemical level to the cellular milieu.

To address this challenge, we have combined somatic cell genetics with single-molecule localization microscopy, and developed bespoke analytical tools, to visualize the molecular intermediates formed during end-resection and RAD51 filament assembly at cellular sites of DNA damage at resolutions approaching ∼30–40 nm (full-width at half-maximum—FWHM, see Methods). We developed analytical methods that enabled us to create detailed molecular maps of RPA and RAD51 localization at radiation-induced damage foci in several human cell types either wild-type or deficient for BRCA2, over time after exposure to DNA damage. Our observations suggest a model for HR in which (a) DNA end-resection and RAD51 filament assembly at each cellular DSB occur concurrently at adjacent but non-overlapping sites, (b) BRC repeats are necessary for initial RAD51 filament formation at sites of damage and (c) RAD51 filament extension is critically dependent on the C-terminal region of BRCA2. Our observations provide fresh insight into the cellular mechanisms underlying HR through the high-resolution mapping of its molecular intermediates using single-molecule localization microscopy.

## MATERIALS AND METHODS

### Cell culture

HeLa Kyoto, HeLa Tet On, HPNE, LN9 and EUFA423 cells were cultured in Dulbecco's modified Eagle's medium (DMEM, Gibco) supplemented with 10% FBS, 100 U/ml penicillin and streptomycin. Cells were maintained at 37 °C with 5% CO_2_. The human BRCA2-deficient fibroblast cell line, EUFA423, and human fibroblast cell line obtained from clinically healthy individuals LN9 was a kind gift from VU University Medical Center (Amsterdam, The Netherlands) (53). The reconstituted cell line (EUFA423 + BRCA2) was generated by introducing FLAG tagged full-length BRCA2 cDNA construct into the human BRCA2-deficient fibroblast cell line EUFA423 cells as described previously (54). BRC4 expressing cell line (HeLa Tet On clone 4.23) was generated using Tet-On Inducible Gene Expression System (Clontech) as described previously (55); this cell line expresses BRC4 repeat (BRCA2 residues 1481–1553), C-terminally tagged with myc epitope and 3xNLS (nuclear localization signal). hTERT-HPNE human ductal pancreatic cell line was purchased from Lgcstandards. Cells were plated in 8-well, glass-bottom (1.5 thickness) ibidi μ-slides (ibidi, 80827).

### DNA damage induction, BrdU labeling and RNA interference

DNA damage was induced by irradiating cells with X-rays to achieve a dose of 5 Gy, utilizing a RS225M Research Cabinet (Xstrahl). After irradiation, cells were allowed to repair DNA damage for 30 min up to 5 h in a standard cell culture incubator.

For BrdU and RPA co-staining experiments, cells were incubated with 10 μM BrdU for 24 h (Sigma, B5002) and washed twice in growing medium prior to DNA damage. BRCA2 knock-down was performed using short interfering (si)RNA reverse transfection according to manufacturer protocol simultaneously with plating cells in ibidi μ-slides with DharmaFECT1 (Dharmacon, UK) transfection reagent. Experiments were performed 48 h after siRNA transfection. Cells expressing siRNA were compared to mock-transfected cells from the same cell culture day. Expression of BRC4 peptide in HeLa Tet On cells was induced with doxycycline (1 μg/ml, Sigma) and compared to control cells from the same culture not treated with any compound.

### Immunocytochemistry

**Figure 1.**
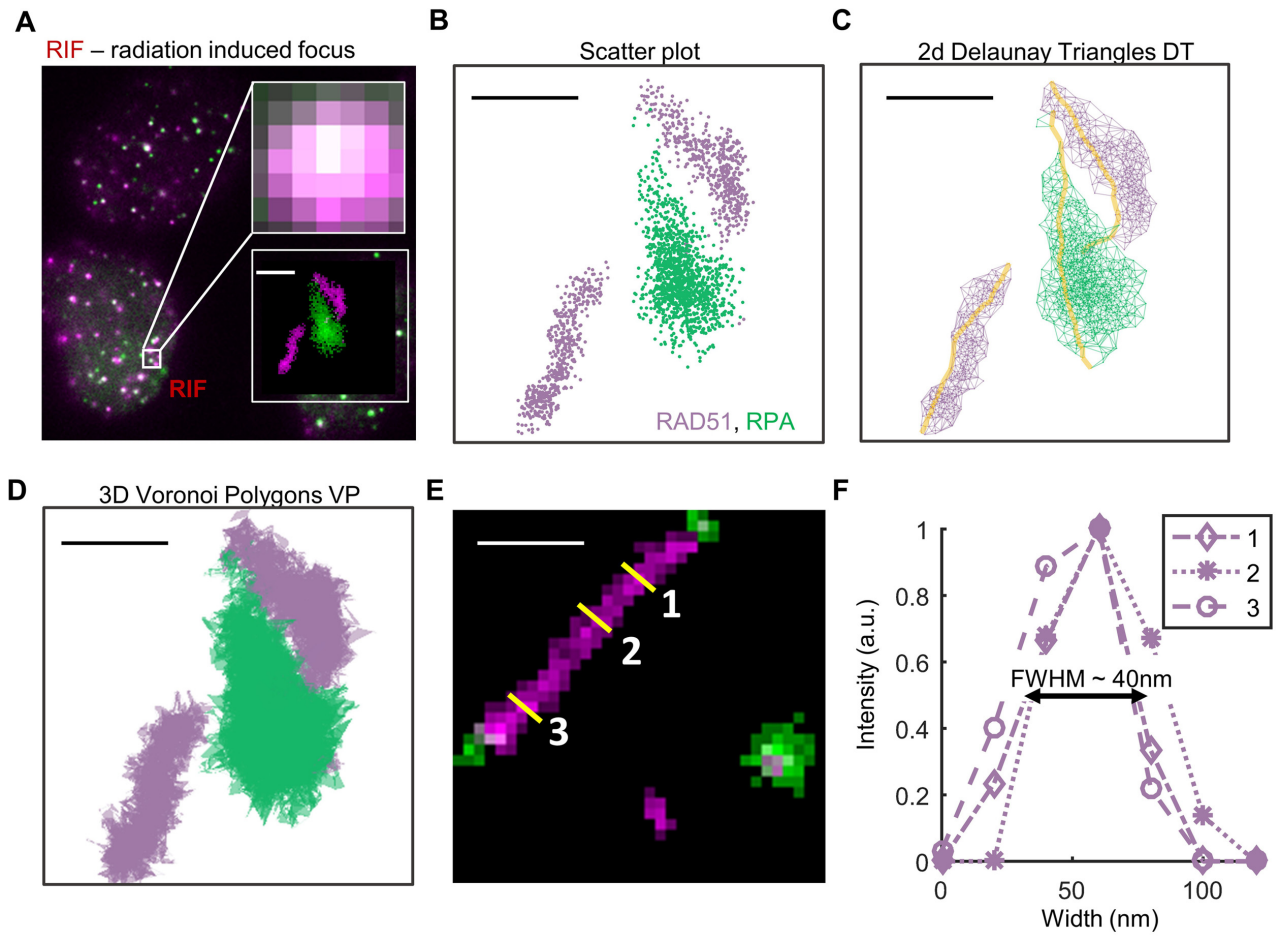
High-resolution molecular maps of cellular DNA damage foci using Voronoi polygons (VP) and Delaunay triangulation (DT). (**A**) Representative HeLa Kyoto cell 5 h after exposure to 5 Gy of X-rays radiation (IR) stained for RAD51 (magenta) and RPA (green). The insets show a magnified region containing a single radiation-induced focus (RIF) as diffraction limited (top; 160 nm pixel size) or d-STORM (bottom; 10 nm pixel size) images. (**B**) Scatter plot of single-molecule localizations of RAD51 (violet) and RPA (green). (**C**) The segmented DT graphs of RAD51 (violet) and RPA (green) were used to compute the extension of structure (maximum shortest path) which is highlighted with yellow lines. (**D**) RAD51 (violet) and RPA (green) visualized with three-dimensional VPs, utilized for image de-noising. (**E**) d-STORM image of a straight RAD51 filament used to estimate resolution as the full width at half maximum (FWHM) of the linear cross-section shown in (**F**). Scale bar—250 nm.

**Figure 2.**
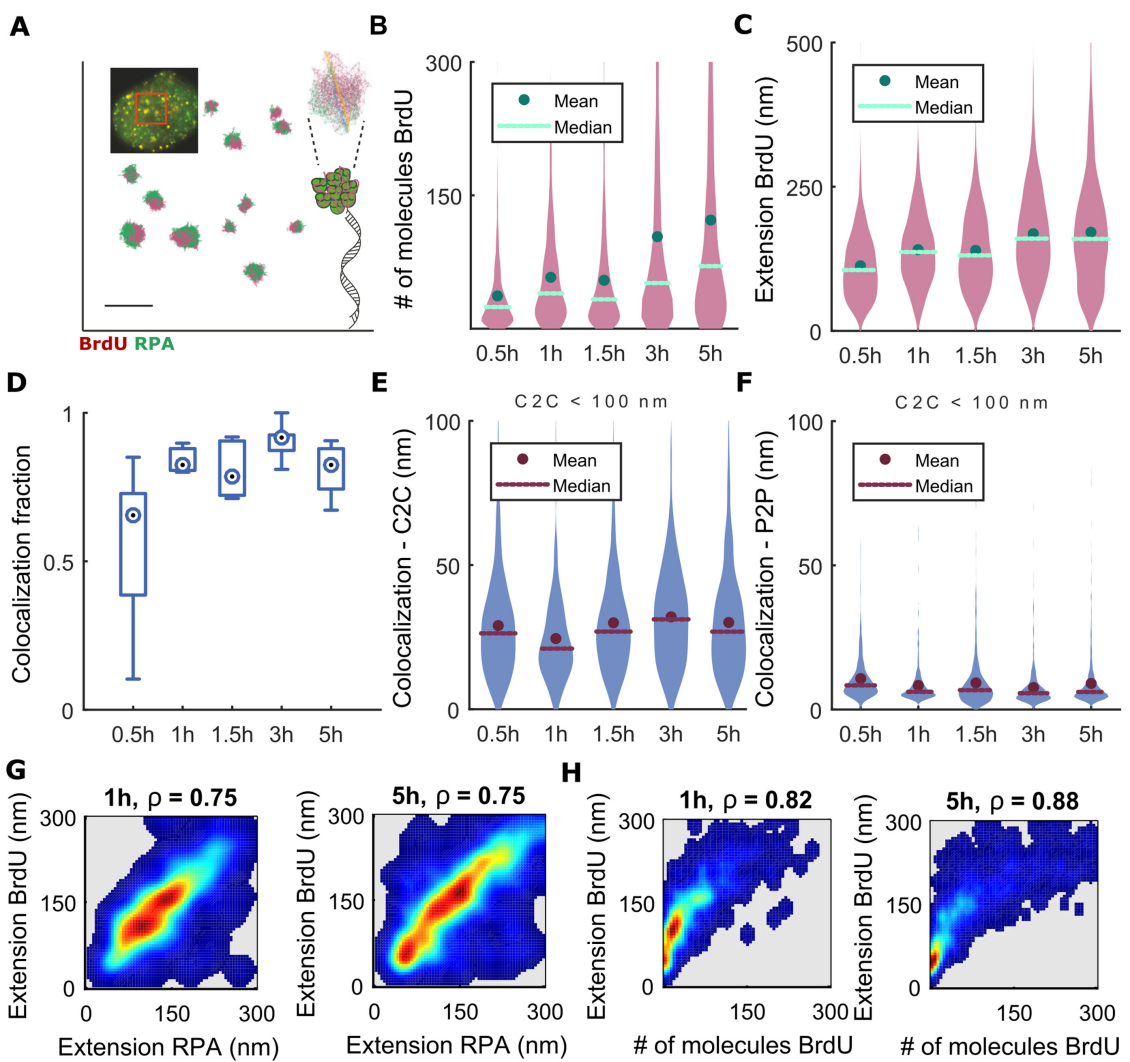
Characterization of DNA resection foci after IR induced DNA damage by BrdU (ssDNA) and RPA staining. (**A**) BrdU (purple) and RPA (green) foci in Hela Kyoto cell 5 h after exposure to 5 Gy IR represented as VP. The top left inset shows a diffraction-limited image and the region of interest (red square) visualized as VP. On the right, the schematic representation of RPA wrapped around ssDNA is shown together with the close-up of a single RIF represented as DT; here, yellow (BrdU) and blue (RPA) lines mark the extension of the graphs. (**B**) Distribution of the number of detected nucleosides inside BrdU clusters. (**C**) Distribution of BrdU cluster extension. (**D**) Fraction of BrdU clusters co-localized with RPA. Distribution of the distances from BrdU cluster centroid to RPA cluster centroid (C2C) that exhibits distance shorter than 100 nm from the nearest RPA cluster centroid, C2C < 100 nm (**E**) or inter-molecular distance (P2P) from BrdU cluster with C2C < 100 nm from the nearest RPA cluster (**F**). (**G**) 2D density plots showing the correlation between the extension of RPA and BrdU clusters at representative time points after DNA damage, 1 h (left) and 5h (right). (**H**) 2D density plots showing the correlation between the number of detections within BrdU clusters and their extension. Here, ρ is the Spearman correlation coefficient. All distributions are computed on the aggregate of two independent biological replicates. The abscissae in panels B–F indicate time after irradiation. The number of BrdU-RPA pairs analysed is 835, 361, 935, 624 and 1008 at 30 min, 1 h, 1.5 h, 3 h and 5 h, respectively. Exact *P*-values for multiple means max-t comparison test are given in Supplementary Figure S5. Scale bar—250 nm.

**Figure 3.**
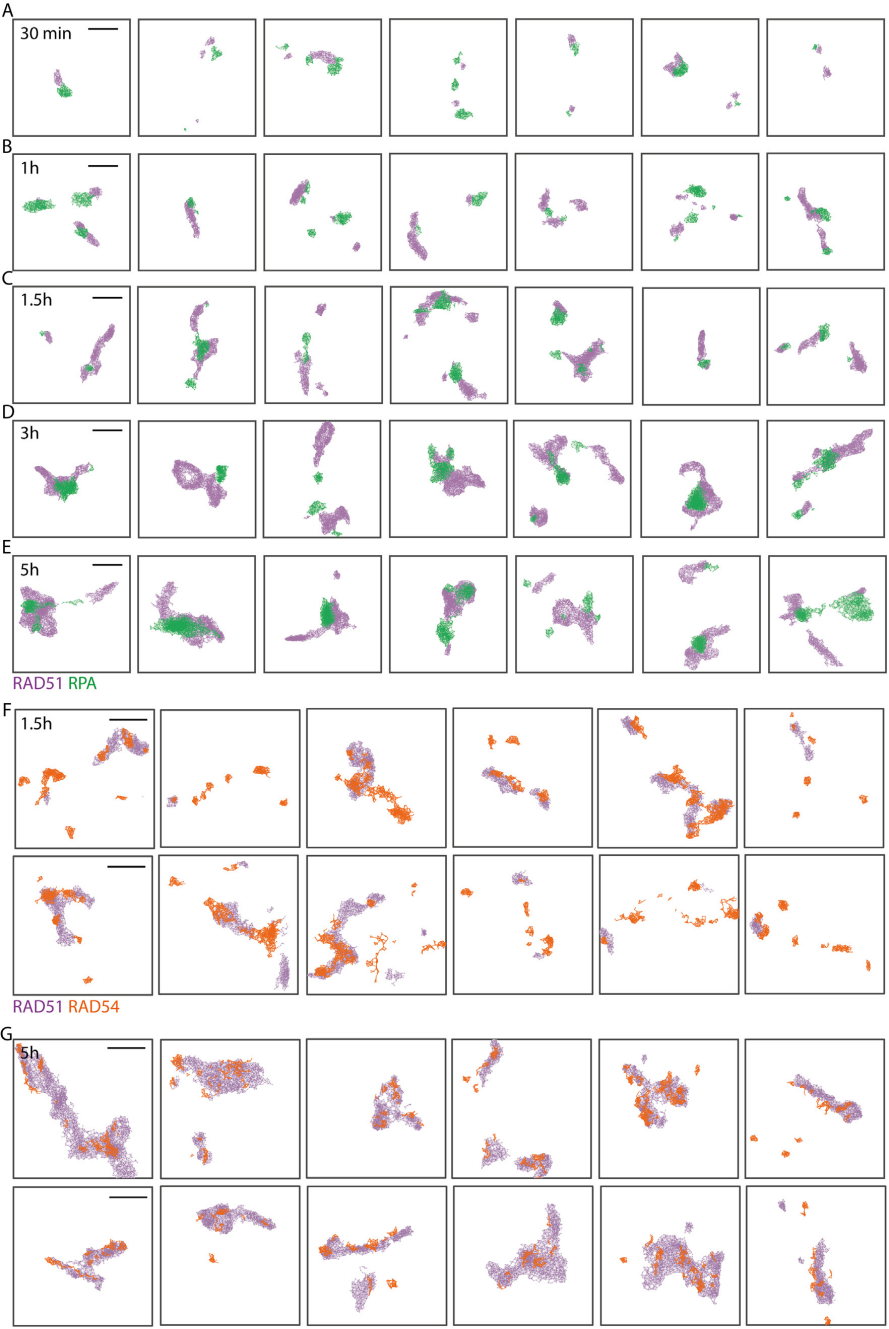
Representative DT visualizations of RAD51 (violet), RPA (green) and RAD54 (orange) molecules at radiation-induced foci in Hela Kyoto cells at 30 min (**A**), 1 h (**B**), 1.5 h (**C, F**), 3 h (**D**) and 5 h (**E, G**) after exposure to 5 Gy IR. Scale bar—250 nm.

**Figure 4.**
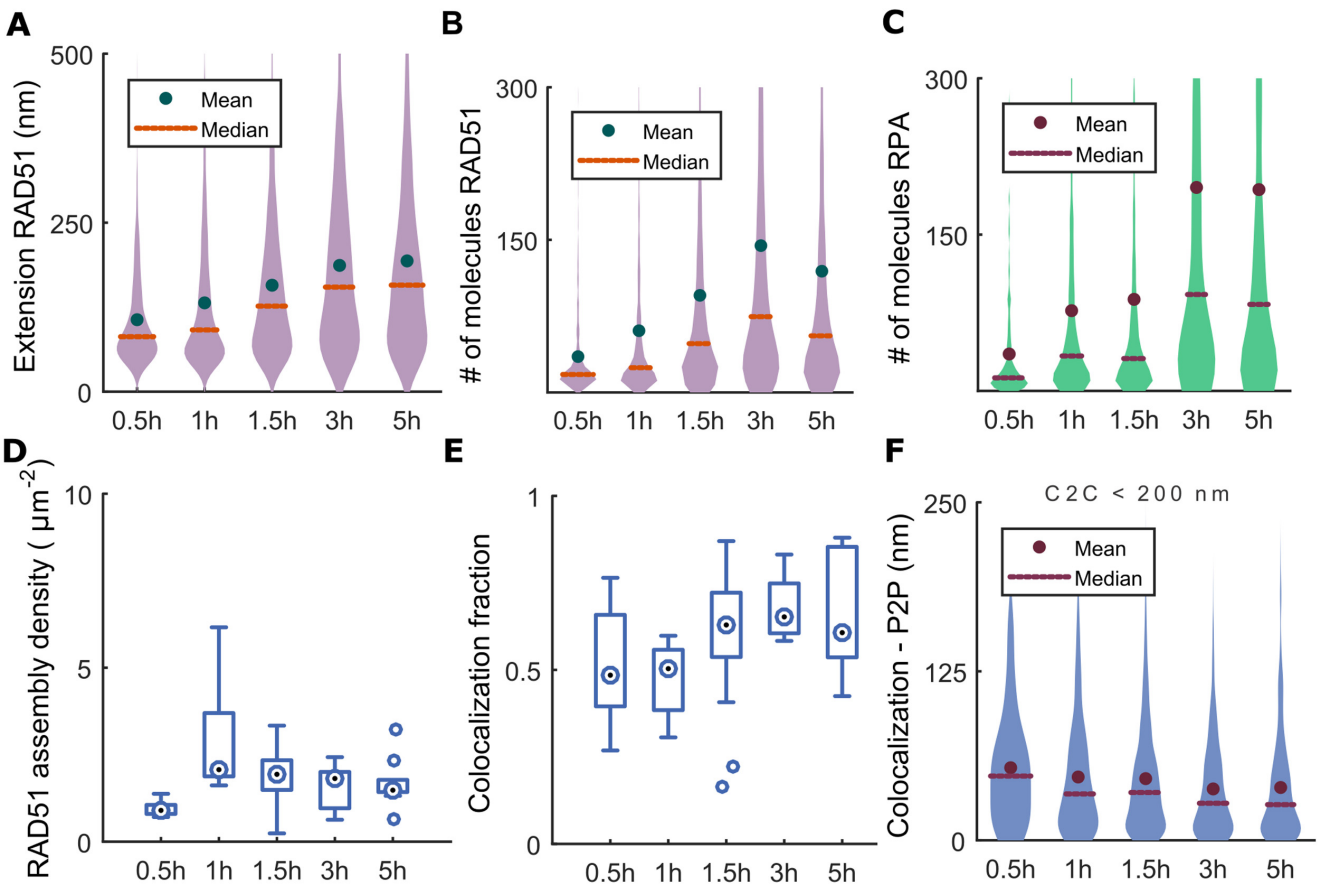
Topography of RAD51 and RPA at radiation-induced foci in Hela Kyoto cells. (**A**) Distribution of the RAD51 cluster length measured by DT graph extension. (**B**) Distribution of the number of RAD51 molecules in a filament. (**C**) Distribution of the number of molecules in RPA cluster. (**D**) RAD51 filament density depicted as structures per surface area. (**E**) Fraction of RAD51 clusters co-localized with RPA. (**F**) Distribution of inter-point distance (P2P) from RAD51 filament to RPA cluster located at centroid-to-centroid (C2C) distance shorter than 200 nm. All distributions are computed on the aggregate of 5 independent biological replicates. Abscissae indicate time after exposure to 5 Gy IR. The number of RAD51–RPA pairs that were analysed is 278, 1148, 2550, 949 and 938 at 30 min, 1 h, 1.5 h, 3 h and 5 h, respectively. Exact *P*-values for multiple means max-*t* comparison test are given in Supplementary Figure S9.

**Figure 5.**
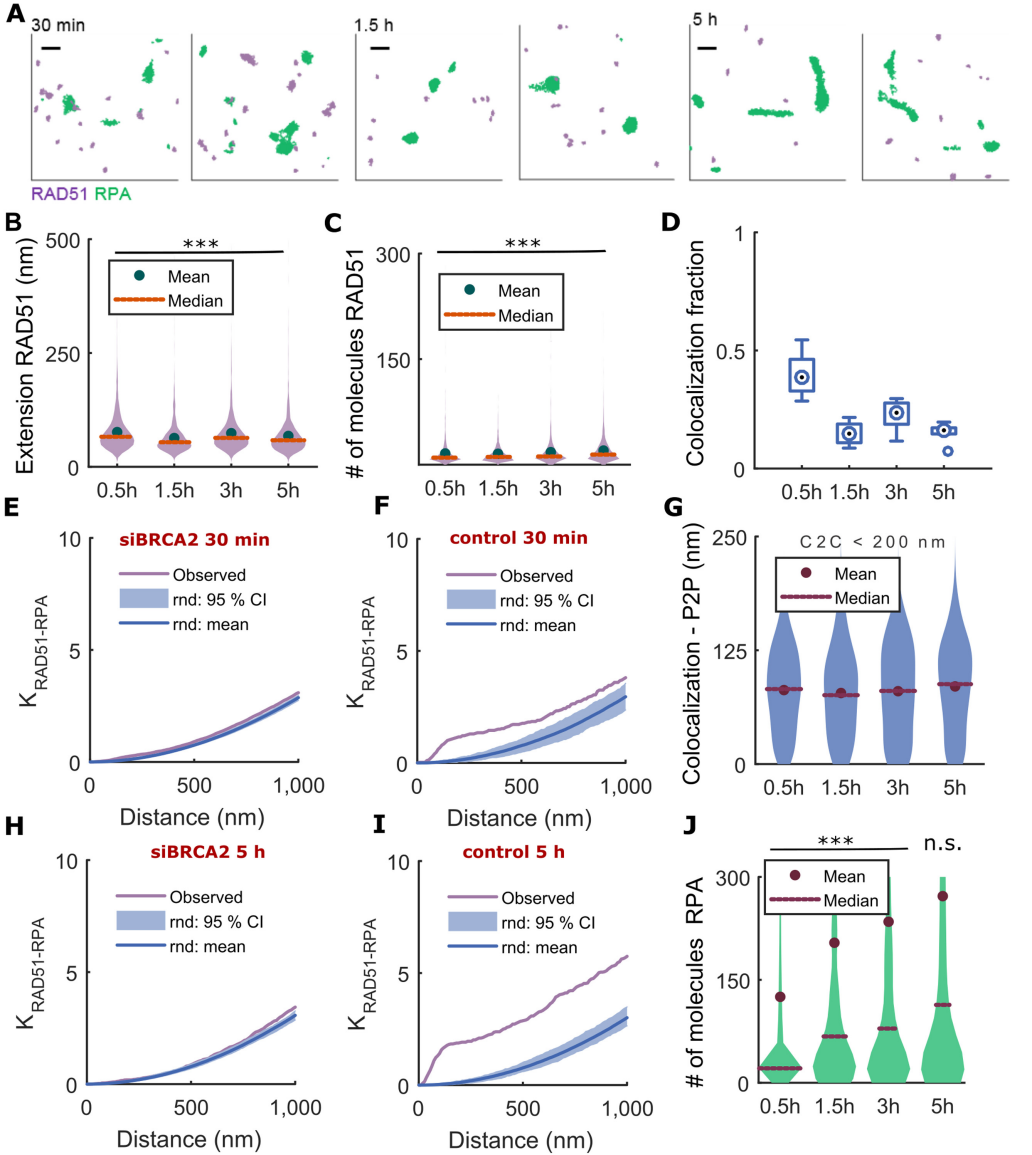
Topography of RAD51 and RPA at radiation-induced foci in Hela Kyoto cells after siRNA-induced depletion of BRCA2. (**A**) RAD51 (purple) and RPA (green) foci represented as DT. (**B**) Distribution of lengths of RAD51 assemblies measured by DT graph extension. (**C**) Distribution of the number of RAD51 molecules inside RAD51 filament. (**D**) Fraction of RAD51 filaments co-localized with RPA. Bivariate Ripley's function for RAD51 and RPA in (**E, H**) siRNA BRCA2 and (**F, I**) mock transfected control cells for 30 min (top) and 5 h (bottom) after exposure to 5 Gy IR. The purple line marks the experimental Ripley's function while the shaded blue areas and the blue lines show the 95% confidence intervals (CI) and mean obtained by repetitive randomization of cluster centroid positions. (**G**) Distribution of inter-point distance (P2P) for every RAD51 assembly to RPA cluster located at centroid-to-centroid (C2C) distance shorter than 200 nm. (**J**) Distribution of the number of molecules inside RPA cluster. Distributions were computed over data aggregated from three independent biological replicates. Abscissae indicate time after exposure to 5 Gy IR. The number of RAD51-RPA pairs that were analysed is 2099, 1283, 1721 and 2153 at 30 min, 1.5 h, and 3–h, respectively. Exact *P*-values for multiple means max-t comparison test are given in Supplementary Figure S13. Stars indicate two sample two-sided Wilcoxon rank sum test between siBRCA2 and matched control cells at a single time, ***P< 0.001, n.s. not significant. Scale bar—250 nm.

**Figure 6.**
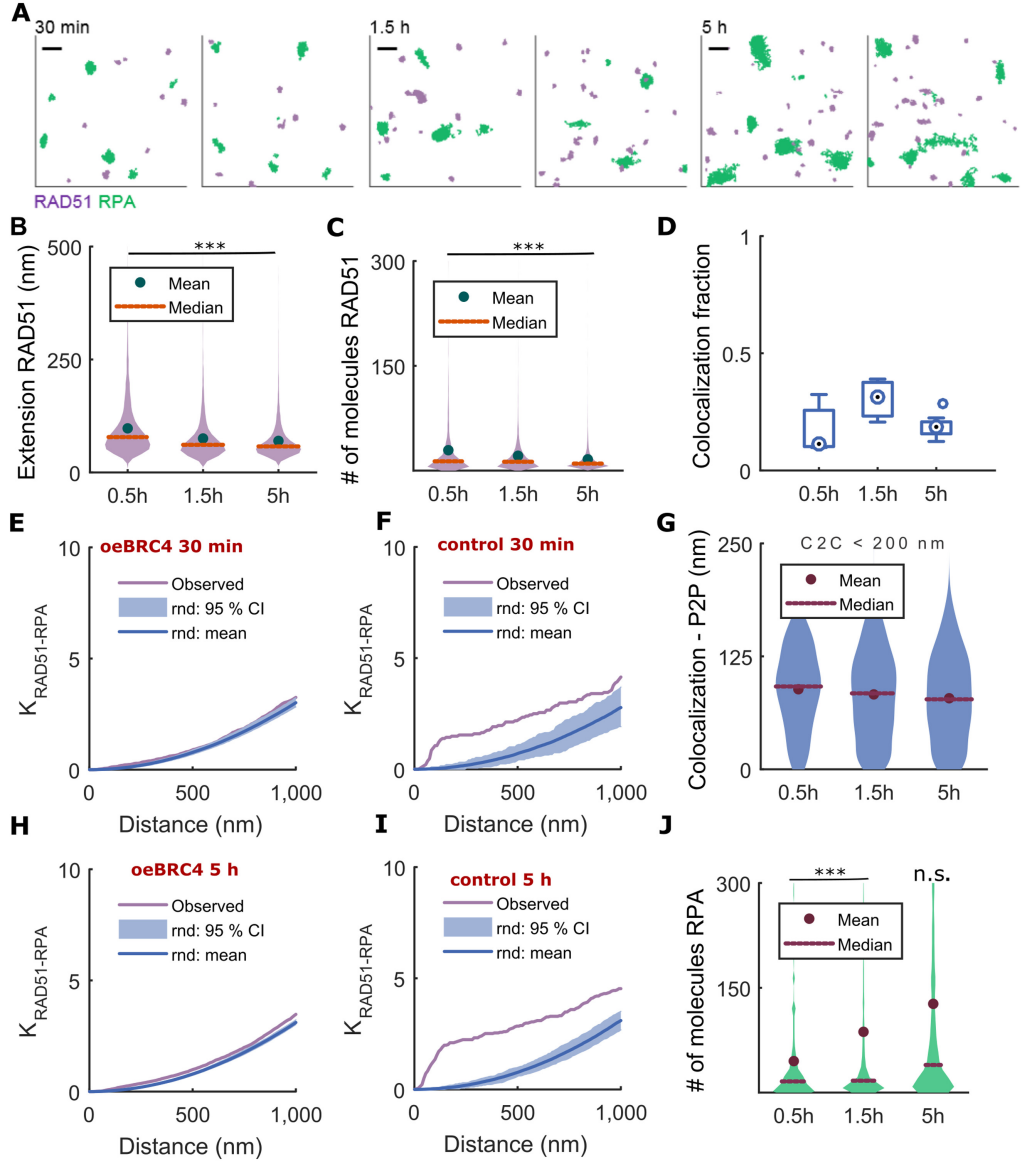
Topography of RAD51 and RPA at radiation-induced foci in Hela Tet ON cells expressing the dominant-negative BRC4 peptide. (**A**) RAD51 (purple) and RPA (green) foci represented as DT. (**B**) Distribution of lengths of RAD51 assemblies measured by DT graph extension. (**C**) Distribution of the number of RAD51 molecules inside RAD51 filament. (**D**) Fraction of RAD51 filaments co-localized with RPA. Bivariate Ripley's function for RAD51 and RPA in (**E, H**) oeBRC4 and (**F, I**) control cells for 30 min (top) and 5 h (bottom) after exposure to 5 Gy IR. The purple line marks the experimental Ripley's function while the shaded blue areas and the blue lines show the 95% confidence intervals (CI) and mean obtained by repetitive randomization of cluster centroid positions. (**G**) Distribution of inter-point distance (P2P) for every RAD51 assembly to RPA cluster located at centroid-to-centroid (C2C) distance shorter than 200 nm. (**J**) Distribution of the number of molecules inside RPA cluster. Distributions were computed over data aggregated from three independent biological replicates. Abscissae indicate time after exposure to 5 Gy IR. The number of RAD51-RPA pairs that were analysed is 2074, 5013 and 4321 at 30 min, 1.5 h and 5 h, respectively. Exact *P*-values for multiple means max-*t* comparison test are given in Supplementary Figure S15. Stars indicate two sample two-sided Wilcoxon rank sum test between oeBRC4 and matched control cells at a single time, ***P< 0.001, n.s. not significant. Scale bar—250 nm.

**Figure 7.**
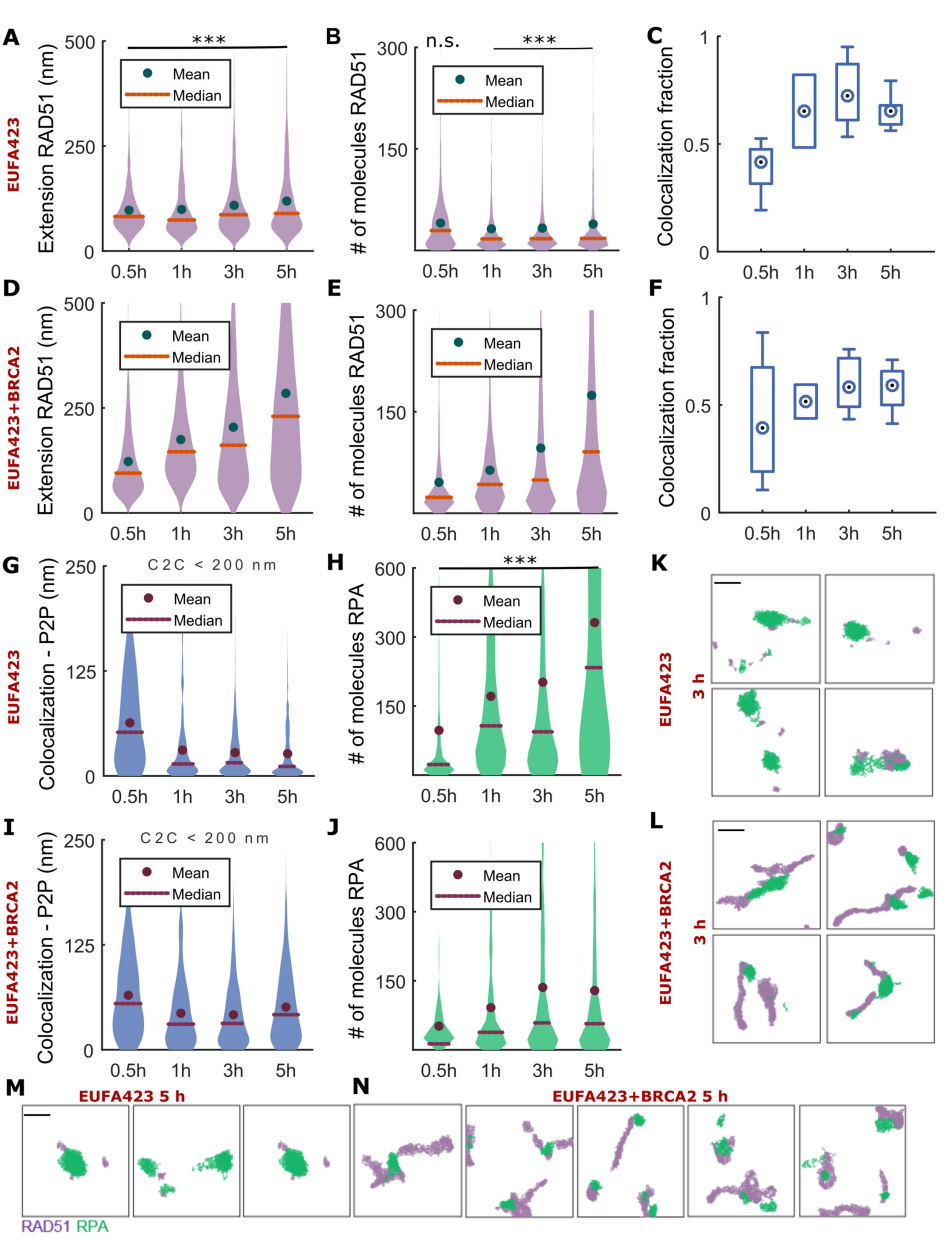
Topography of RAD51 and RPA at radiation-induced foci in EUFA423 and reconstituted EUFA423 + BRCA2 cells. Distribution of the RAD51 filament length measured by DT graph extension in (**A**) EUFA423 and (**D**) EUFA423 + BRCA2. Distribution of the number of RAD51 molecules inside RAD51 filament in EUFA423 (**B**) and EUFA423+BRCA2 (**E**). Fraction of RAD51 clusters co-localized with RPA in (**C**) EUFA423 and (**F**) EUFA423+BRCA2. Distribution of inter-point distance (P2P) from RAD51 filament to RPA cluster located at centroid-to-centroid (C2C) distances shorter than 200 nm in (**G**) EUFA423 and (**I**) EUFA423 + BRCA2 cells. Distribution of the number of RPA molecules inside the cluster in (**H**) EUFA423 and (**J**) EUFA423 + BRCA2 cells. Typical RAD51 filaments (violet) and RPA clusters (green) in EUFA423 + BRCA2 and EUFA423 at (**K, L**) 3 h and (**M, N**) 5 h after exposure to 5 Gy IR. Distributions were computed over data aggregated from five independent biological replicates. Abscissae indicate time after exposure to 5 Gy IR. The number of RAD51-RPA pairs that were analysed (EUFA423 + BRCA2/EUFA423) is 1352/1384, 382/559, 1119/1157 and 1158/920 at 30 min, 1 h 3h and 5 h, respectively. Exact *P*-values for multiple means max-t comparison test are given in Supplementary Figure S17. Stars indicate two sample two-sided Wilcoxon rank sum test between EUFA423 and EUFA423 + BRCA2 at a single time, ***P< 0.001, n.s. not significant. Scale bar—250 nm.

Cells were washed on ice once with phosphate-buffered saline solution PBS followed by fixation in 2% formaldehyde (Agar Scientific) at room temperature (RT) for 20 min, then washed three times in PBS. To quench remaining aldehydes cells were incubated with 50 mM NH_4_Cl (Sigma) for 30 min at RT. Next, cells were permeabilized with 0.2% Triton X-100 (Fisher Chemical) for 10 min at RT. Non-specific protein-protein interactions were blocked with 2% bovine serum albumin, 0.1% Triton X-100 and 0.05% Tween (NBS Biological) in PBS for 1 h. Primary antibodies were incubated in blocking solution at 4°C overnight in a humidified chamber. In Figures 1, 3–5 and 7, we used rabbit anti-RAD51 monoclonal antibody (Abcam, ab 133534) at a 1:2000 dilution; mouse anti-RPA32 monoclonal antibody (Abcam, clone 9H8, ab2175) at 1:250. In Figure 6, we used mouse anti-myc tag (Santa Cruz, clone 9E10) at 1:250, and rabbit anti-RAD54 polyclonal antibody (H-152, Santa-Cruz) at 1:100. In Figure 2, we used mouse anti-BrdU (Bromodeoxyuridine, Sigma) monoclonal antibody (BD Biosciences, clone B44) at 1:100 and rat-anti-RPA32 monoclonal antibody (Cell Signaling, clone 4E4, ab2208) at 1:500; RAD51 was fluorescently stained with goat anti-rabbit (Invitrogen, A-21246) Fab2 secondary antibody fragment conjugated to Alexa Fluor 647. RPA was detected with goat anti-mouse (Sigma, SAB4600400) CF568 conjugated Fab2 secondary antibody fragment or goat anti-rat (Sigma, SAB4600086) with CF568 conjugated secondary antibody. BrdU was detected in non-denaturating conditions with goat anti-mouse (Stratech Scientific, 115-607-003-JIR) Fab2 secondary antibody fragment conjugated to Alexa Fluor 647. Myc tag was revealed with goat anti-mouse Alexa 488 secondary antibody (Sigma, SAB4600387). RAD54 was detected with goat anti-rabbit (Sigma, SAB4600310) CF568 conjugated Fab2 secondary antibody fragment.

### Direct stochastic optical reconstruction microscopy (d-STORM)

Samples were prepared for d-STORM utilizing a buffer containing 100 mM MEA-HCL (Sigma, M6500), 10% glucose (Sigma), 0.5 mg/ml glucose oxidase (Sigma, G2133) and 40 μg/ml catalase (Sigma, C100) in water at pH 7.5. Samples were imaged at room temperature in sealed 8-well ibidi μ-slides by direct STORM on an inverted N-STROM microscope (Nikon Ti, Japan) with a an Apochromat 100×/1.49 NA oil immersion objective. In order to reduce thermal drifts, samples mounted on the microscope were let to equilibrate for at least 30 minutes before imaging. Images were then acquired in highly inclined illumination mode and focal drift was prevented with hardware autofocusing (Nikon Perfect Focus System). CF568 and Alexa Fluor 647 were first pumped in their dark state using the 640 nm (∼150 mW) and 561 nm (∼90 mW) laser lines at maximum laser intensity and then continuously acquired at ∼3 kW/cm^2^. All images presented are 2D projections of d-STORM images that were acquired utilizing astigmatism-based 3D STORM. Z-calibration was performed using 100 nm multicolor fluorescent microbeads (Tetraspeck, Invitrogen) according to Nikon N-STORM manual. Data was acquired in ‘streaming mode’ with a field-of-view FOV 256 × 256 pixels (160 nm pixel size), at 65 frames per second for 25 000 frames with an EMCCD camera (iXon Ultra DU897, Andor). The sparsity of single molecules per frame was controlled with 405 nm laser (∼30 mW) at ∼2% of total power. CF568 was always acquired sequentially to Alexa Fluor 647, utilizing a Quad Band Set for TIRF applications (Chroma, TRF89901, ET—405/488/561/640 nm) and the ET600/50 nm and ET645/75 m for 647 emission filters (Chroma) to reduce cross-talk between the detection of the two fluorophores.

### Single molecule data analysis

Single molecule localization analysis was performed with the Nikon N-STORM software setting the maximum possible width of a spot to 700 nm and the maximum axial ratio (ratio of elongation in X- and Y-directions) to 2.5. Multicolor fluorescent microbeads were used as fiducial markers to register the two channels. Z-position calibration and chromatic aberrations corrections were performed using the same fluorescence microbeads sample (Supplementary Figure S3C). Lateral drift was automatically corrected using a correlation algorithm on molecular locations between successive frames. Lateral localization precision was calculated using Thompson equation (56) for *x* and *y* dimension and combine lateral localization precision was computed as σ = √(σ_*x*_^2^ + σ_*y*_^2^). The median value for single molecule localization precision (57) was ∼7 and ∼10 nm for Alexa Fluor 647 and CF568, respectively (Supplementary Figure S2A and B). To correct for multiple localization of the same fluorophore, molecules detected in up to 10 consecutive frames were considered as a single molecule detection. Resolution, the smallest resolvable distance between two structures, was estimated using the data shown in (Supplementary Figure S2). Resolution in single molecule localization-based microscopy depends primarily on single emitter localization precision and sampling density. We have estimated 2D and 3D sampling density using volume (3D) and area (2D) of Voronoi polygon (see next section) as well as inter-molecular nearest neighbor pairwise distances (Supplementary Figure S2H, I and J respectively) in both 2D and 3D space. However, the size of structures reported was estimated from 2D projections of 3D STORM data and 3D Voronoi diagrams were only used for data denoising step. As proposed in *Deschout et al.*, we combined localization precision, ∼10 nm (Supplementary Figure S2A and B), 2D median inter-molecular distances (an analogue for Nyquist criterion), ∼5 nm, (Supplementary Figure S2I and J) and size of tagged antibodies (∼15 nm) to estimate the resolution (∼30 nm) (57). However, such estimation may not correct for other factors influencing the resolution, e.g.: antibody steric hindrance, not fully corrected optical aberrations, antibody thermal movement. To account for these factors, we plotted cross-sectional profiles of straight RAD51 filaments and obtained ∼40 nm FWHM (Figure 1E and F), a well accepted empirical estimate of lateral resolution that confirms the high resolution we achieved.

### Cluster data analysis

The remainder of the data analyses were performed using a custom Matlab programme (Mathworks, UK) that has been deposited in the GitHub repository (https://github.com/inatamara/Grafeo-dSTORM-analysis-). First, all localizations with <1000 detected photons or localization precision lower than 15 nm were discarded (Supplementary Figure S2A–C). This effective de-noising step was followed by segmentation based on 3D Voronoi diagrams (VD; Supplementary Figure S1B) (58). Shortly, localizations were used as seeds (S_i_) to partition 3D space with an *i*th Voronoi polygon VP_*i*_ so that each point within polygon VP_*i*_ is closer to the seed S_*i*_ than any other seed/localization. Therefore, each localization has a corresponding VP which size depends on the local density of localized molecules. The denser the localizations, the smaller the VP (Supplementary Figure S1B) thus permitting us to discriminate with a simple threshold on the size of Voronoi polygons (Supplementary Figures S1C and S2E, H, I) specific detection of protein clusters (e.g. RAD51 filaments) compared by sparse, random, localization arising non-specific staining that represent background noise. Initially, all VPs larger than the median value (typically ∼10^−5^ nm^3^ and ∼5 × 10^−6^ nm^3^ for CF568 and Alexa Fluor 647, respectively; see Supplementary Figure S2H–J) within an experimental dataset were discarded. Following this second de-noising step, the two channels were aligned by minimizing 3D Euclidean distance between fiducial markers. Finally, small isolated detections were suppressed by thresholding conditional bivariate and univariate distance distribution function (Supplementary Figure S2F, G): a given localization was typically rejected unless it had at least 30 or 10 neighboring detections of the same color or different color, respectively, within a 100–150 nm radius. This radius correspond to the sum of the mean value and three standard deviations of the typical distribution of inter-molecular distances detected (Supplementary Figure S2F and G).

Threshold values on the number of neighboring detections corresponded to the average number of detection obtained by imaging isolated conjugated secondary antibodies on the glass in the same imaging conditions. In summary, rejection of detected molecules based on Voronoi polygons and statistical analysis of localizations provided an efficient rejection of non-specific staining or isolated, non-chromatin bound, proteins labeled with—on average—a single secondary antibody (RAD51 and RPA antibodies were monoclonal). Next, 2D Delaunay triangulation (DT) was computed (59) (Supplementary Figure S1D). Detections were assigned to discreet clusters, connected components, by removing all edges larger than 30 nm from DTs, i.e. the median of typical edge length distribution. DT edge thresholding permitted us to disconnect closely spaced structures that are connected by longer edges than intra-cluster edges (Supplementary Figure S1D–G), thus providing a robust methodology for segmentation of molecular clusters. All the parameters were fixed throughout analyses, except the threshold on Voronoi polygon volumes that was occasionally decreased in the presence of higher background or too many microbeads (fiduciary marks) that may skew the analyzed distributions; however, this value was kept between mean and median of Voronoi polygon volume distributions. Above steps allowed for efficient de-noising, filtering and cluster segmentation as exemplified in Supplementary Figure S3A and B. The size of each cluster was estimated by means of the number of detected molecules within each cluster. The number of molecules was estimated by dividing the number of localization within a cluster by expected number of localization obtained from isolated secondary antibodies used to label corresponding proteins (Supplementary Figure S2D). Equivalently, the size of a cluster was given as extension—the maximum shortest paths spanning the graph (Figure 1B, Supplementary Figure S1H). We report both measures, as each one will have limitation; e.g. because of 2D projection, size of a globular, compact cluster measured by extension display smaller incremental changes and saturates faster than number of detection (Figure 2H). However, number of detections is sensitive to the multiple counts of the same fluorophore as well as the size of image stack acquired.

Proximity between structures tagged with Alexa Fluor 647 and CF568 was estimated in two alternative ways: as the distance between weighted (by number of photons) centroids of two clusters (centroid-to-centroid or C2C) or as the median of the first nearest neighbor inter-point distances between every detection in one cluster to the closest detection in the other clusters (point-to-point or P2P). An estimate for the proximity of RAD51 and RPA clusters was also estimated by the distance between the end of RAD51 filament and the closest detection in neighboring RPA cluster (end-to-point or E2P); the ends of RAD51 filaments were determined as the two points connected by the extension path defined earlier (node 1 and 35, Supplementary Figure S1H). The distributions of these estimations of size and co-localization are shown as bean plots overlaid with mean and median values. Co-localization fraction was calculated per cell as a fraction of RAD51 filaments (BrdU clusters) having RPA at the C2C < 200 nm (<100 nm).

The Bivariate Ripley's function was used to validate RAD51-RPA proximity and it was calculated for each cell separately using equation (12) in (60). Ripley's function obtained on experimental data was compared to randomized data, obtained by assigning random position to the centroid of a cluster enclosed by boundary of the nucleus. The 95% confidence intervals (CI) were determined by repeating randomization of cluster localization 100 times, and evaluating Ripley's function, at each step. If the experimental Ripley's curve lies above, below or within 95% confidence intervals, this suggests clustering, exclusion or independent distribution, respectively.

### Statistical analyses

Simultaneous comparison of the mean values of multiple group was performed using max-t two-tailed test using multcomp R package (61). This framework was designed as an alternative to ANOVA or Kruskal–Wallis tests as a more robust method for comparing data characterized by unbalanced group size, non-normality and heteroscedasticity. Prior to test, data was log-transformed to reduce variance variability and distribution shape difference between groups (compare Supplementary Figure S4B and C). All the statistical tests were performed at significance level alpha = 0.05 and familywise error rate was corrected by adjusting *P*-values using Tukey–Kramer method. Exact *P*-values were shown in figures supplements. Spearman correlation coefficient was used to report non-linear correlations between two variables. Biological replicates are defined as independent experimental preparation including cell culture, treatment, and immunocytochemistry. For every biological replicate we run typically two to three technical replicates performed on the same cell culture day, but with different sample preparation. Comparison of two groups was performed with two sample two-sided Wilcoxon rank sum test.

## RESULTS

### Analysis of damage-induced localization of RPA and RAD51

Cellular sites of DNA damage repair are too small to be resolved with diffraction-limited microscopy techniques that enable a spatial resolution of ∼200 nm. Therefore, we have used direct STochastic Optical Reconstruction Microscopy (d-STORM), a well-established super-resolution optical nanoscopy technique, to dissect cellular events leading to HR. d-STORM is a powerful tool to study molecular complexes inside fixed cells at resolution approaching tens of nanometers (62–66). d-STORM provides coordinates of molecular localization, but usually d-STORM data are represented as pixelated images, often limiting analysis of the spatial organization of the molecules being visualized. This limitation has spurred the development of analytical methods based on molecular coordinates (58,67,68), such as Voronoi diagrams (VD) and Delaunay triangulation (DT), which represent single molecule localisation in a pointillist manner in the 2D Euclidean space. Here, we have developed analytical tools based on VD and DT (see Methods for details and Supplementary Figures S1 and S2), de-noising, foci structural analyses (e.g. clustering, co-localization, size), in a computationally efficient and semi-automated fashion.

Equipped with these tools, we first analyzed the localization of RPA and RAD51 in the nuclei of HeLa Kyoto cells at different times—from 30 min to 5 h—after exposure to 5 Gy ionizing radiation (IR), at which time cells were >90% viable (see Supplementary Figure S6B and C for cell viability and normalized number of RAD51 foci between 30 min and 24 h). Although HeLa represents a transformed human cell line, it has been widely used as a model system for the cellular DNA damage response, after similar IR exposure (69–73). Nevertheless, we note at the outset that the topography and kinetics of RAD51 filament assembly are also similar in other human cell types, which are shown in subsequent figures.

An example image (Figure 1A) obtained by highly inclined illumination microscopy of HeLa Kyoto cells demonstrates RPA (green) and RAD51 (magenta) assemble into microscopic radiation induced damage foci (RIF) in the nuclei of cells 5 h after DNA damage. The inset (boxed) represents the close-up of a single focus as a conventional image (top) and a d-STORM image (bottom). Alternatively, in a representative single damage focus, d-STORM data can be visualized as a scatter plot of RPA and RAD51 localizations (Figure 1B). Super-resolution images (Figure 1A and B) show a cluster of RPA molecules (green) abutting an extended filament formed by RAD51 molecules (violet). Scatter data can be assigned to discreet graphs using DT (Figure 1C and Supplementary Figure S1D–G). This approach enables the measurement of the diameter of globular clusters or the length of extended filaments, by calculating the maximum shortest path spanning the graph, depicted as yellow lines (Figure 1C) (see Materials and Methods and Supplementary Figure S1H). Three-dimensional Voronoi polygons (Figure 1D), here used only for image de-noising, can also be used for final visualization of d-STORM data.

The analytical tools we have adapted for the quantitative description of the spatial organization of RPA and RAD51 molecules at the site of DNA damage permit us to achieve high spatial resolutions (illustrated in Supplementary Figures S1 and S2). Figure 1E shows one instance of a straight RAD51 filament that can be used to estimate the achieved resolution empirically, confirming a 30–40 nm FWHM resolution value (Figure 1F); this value accounts for all those experimental features (e.g. antibody size, incomplete drift correction, spherical aberrations) that can deteriorate actual spatial resolution relative to theoretical limits.

### Kinetics and topography of ssDNA and RPA accumulation

Biochemical studies—supported indirectly by genetic analyses—suggest that in somatic human cells HR is initiated when DNA ends at double strand breaks (DSBs) are resected to expose overhanging 3′-ended ssDNA, then quickly coated by RPA (9). *In vitro* assays have shown that the displacement of RPA by RAD51 and the subsequent assembly of an ordered helical nucleoprotein filament encasing ssDNA are critical steps for subsequent events leading to HR. These *in vitro* data suggest the existence of either extended (74) or condensed (75,76) RPA-coated ssDNA structures that are progressively dissociated by RAD51 loading. To determine what occurs in cells, we co-stained IR-exposed cells for RPA, along with ssDNA molecules detected by 5-bromo-2′-deoxyuridine (BrdU) labeling and antibody staining under non-denaturing conditions (19).

Curiously, in intact cells, super-resolution data show that resection foci of RPA and ssDNA marking end-resection localize in coincident, globular, compact clusters and rarely assume an extended conformation (Figure 2A). These observations provide a first line of evidence that RPA-coated ssDNA behaves in cells like condensed or collapsed polymers, as previously proposed from *in vitro* data (76). Moreover, the size of globular BrdU and RPA clusters increases with time over hours, suggesting that DNA end-resection occurs throughout HR, and not merely before RAD51 loading. To confirm this inference, we analyzed changes in the size of clusters containing BrdU-labeled ssDNA over time. Figure 2B and C shows distributions (bean plots) of the number of BrdU nucleotides per cluster and the length of clusters, respectively. The number of BrdU nucleosides was estimated by dividing the number of localized molecules inside the BrdU cluster, by the median number of detections obtained from individual isolated fluorophore-conjugated antibodies (see Methods). The large majority of the clusters (>90%) contains <80 detected BrdU nucleotides in ssDNA, 30 min after irradiation (mean ∼35); subsequently, cluster size increases reaching a mean of ∼100 and ∼120 detected nucleosides at 3 and 5 h, respectively, with the largest clusters exceeding 250 BrdU-labeled ssDNA nucleosides.

Similar conclusions are derived by the analysis of BrdU extension (Figure 2C) and RPA (Supplementary Figures S4A–D and S5A–D). The BrdU cluster size appears to decrease temporarily at 1.5 h post IR for all measurements, albeit not to a statistically significant extent, as indicated by adjusted *P*-values of 0.29 and 0.91 for the number of detections or extension, respectively, and alpha = 0.05 for the multiple means comparison max-t test (see Materials and Methods). However, this observation is consistent with analyses of RPA localization and extension (Supplementary Figures S4C, D and S5A–D), suggesting that the period lasting up to 1.5 h after damage may represent a phase when IR-induced DSBs are partially repaired. We confirmed that RPA co-localizes with BrdU-labeled ssDNA by estimating the fraction of co-localized RPA and BrdU clusters (Figure 2D): more than 90% of BrdU nucleotides co-localize (the distance between centroids of two clusters is <100 nm) with RPA at any given time, with the exception of the time-point at 30 min after damage. However, at this early stage, only very small clusters are detected, with their sizes similar to background noise (arising from non-specific staining and non-chromatin-bound proteins).

We reach similar conclusions with the analysis of the distributions of centroid-to-centroid distance (C2C) (Figure 2E; see Materials and Methods), which for C2C < 100 nm (i.e. co-localizing BrdU–RPA pairs, see Supplementary Figure S4G) is ∼25 nm. We also calculated a bivariate inter-molecular distances (point-to-point, or P2P; see Materials and Methods) (68) between BrdU-labeled ssDNA and RPA (Figure 2F and Supplementary Figure S4E and F). The median inter-molecular P2P distance between BrdU and RPA is in the range of ∼10 nm at any given time, again demonstrating the coincidence between these molecular species. Indeed, if we constrain the analysis to RPA–BrdU pairs lying at centroid-to-centroid distance C2C < 100 nm, then the derived median inter-molecular distance is ∼6 nm (Figure 2F). Furthermore, the number of BrdU and RPA molecules, and the extension of BrdU and RPA clusters, are highly correlated and increase together over time as shown by two-dimensional distributions of these measures (Figure 2G and H) and high Spearman correlation coefficients (Supplementary Figure S5E). These findings demonstrate that BrdU-labeled ssDNA is coated by RPA. However, we do not observe BrdU-labeled ssDNA that is coated by RAD51, which suggests that the BrdU epitope may be masked from antibody recognition in the RAD51-ssDNA complex, consistent with the atomic structure of the RAD51-ssDNA filament recently resolved by cryo-electron microscopy (23,25,26).

### Analysis of RAD51 assembly defines the kinetics and topography of initiation and filament extension

We next ascertained the kinetics and topography of RAD51 assembly, and its relationship to RPA, after DNA damage. Figure 3 shows representative 2D DT graphs of RPA (green) and RAD51 (violet) molecules from 30 min to 5 h after DNA damage. We detected discernible RAD51 focalization only 20–30 min after irradiation (see Supplementary Figure S6A for representative low-resolution images and S7 for visualization of 3D VP on xy, xz, yz planes). These initial small RAD51 clusters abut RPA staining (Figure 3A), but do not overlap. Substantial elongation of RAD51 assemblies into filamentous structures occurs (Figure 3B and C) at later times (≥1 h). The median length (±median absolute deviation) of RAD51 filaments progressively grows from 91 ± 41 nm at 1 h to 127 ± 69 nm (1.5 h), 155 ± 81 nm (3 h) and 158 ± 80 nm by 5 h (Figures 3D, E and 4A). The length of RAD51 filaments is statistically different between every time points (max-*t* multiple means comparison test, alpha = 0.05, *P*-values <0.005), except at 5 h suggesting that maximal filament length is reached between 3 and 5 h (Supplementary Figure S9). The estimated number of RAD51 molecules in the filament reaches the maximum value at 3 h and subsequently significantly decreases at 5 h, suggesting the possibility that repair is gradually completed and RAD51 filament disassembly begins, over this period (Figure 4B), consistent with our findings using conventional microscopy (Supplementary Figure S6C).

We observed that earlier (1–1.5 h) RAD51 filaments tend to be straight, whereas at 3–5 h RAD51 filaments exhibit coiling. Moreover, the apparent absence of a decrease in RAD51 filament extension between 3 and 5 h compared to the number of molecules (Figure 4A and B) may be explained by the appearance of coiled filaments, whose length is likely underestimated by our measurement method.

Notably, similar dynamics and topography of RAD51 assembly, as well as the appearance of filament coiling, are also observed in non-cancerous human ductal pancreatic cell line HPNE (Supplementary Figure S10A–C), suggesting that our findings are not specific to the HeLa model.

RAD51 filament coiling may be caused by changes in filament composition owing to the recruitment of RAD54 (52) or other factors. RAD54 can translocate along DNA and has ATPase-dependent chromatin remodeling activity; it facilitates strand invasion and D-loop resolution, and may help translocate target dsDNA. Moreover, biochemical and cytological studies suggest that RAD54 has a role in RAD51 filament growth (reviewed in (77)). We therefore tested whether RAD54 localization changes during the transition between straight and coiled filaments between 1.5 h and 5 h, but observed no difference. (Figure 3F and G, Supplementary Figure S11A and B).

However, at the resolution we achieved, RAD54 frequently overlaps with RAD51 filaments, forming small interspersed clusters along the filament at 1.5 and 3 h post-damage. Interestingly, RAD54 puncta may precede RAD51 localization (Figure 3F). These observations are consistent with multiple functions for RAD54 during HR, and raise interesting questions for future investigation.

### Relationship between RAD51 and RPA topography

Strikingly, and in contrast to RAD54 localisation, we observe that RPA clusters frequently abut the ends of RAD51 filaments (e.g. see Figure 3 and Supplementary Figure S7 for the 2D visualization of 3D VP). Importantly, RAD51 gradually elongate to form extended filaments, while RPA forms globular clusters indicating that they are bound to different DNA substrates. Both RAD51 filaments and RPA clusters extend over time (Figure 4A–C). However, whilst RAD51 filaments grow steadily between 30 min and 3 h, the number of RPA molecules inside cluster exhibits an apparent pause at 1.5 h (Figure 4C, Supplementary Figures S8 and S9), similarly to BrdU-labeled ssDNA, again suggesting an early phase when DNA damage is partially repaired. However, other effects, like conformation changes, may be responsible for such effect. Similarly to RAD51, the number of molecules within RPA clusters decreases after 3 h (Figure 4C), consistent with the gradual completion of repair.

At 1.5 h, the density of RAD51 assemblies begins to decrease slightly, but not significantly, (Figure 4D) from a peak density of ∼2 RAD51 filaments per squared micrometer. This observation may be interpreted as the gradual completion of RAD51-mediated HR. Similar to BrdU-RPA detection, we attribute the very low density of RAD51 filaments measured at 30 min to difficulties in detecting small clusters. A small RAD51 nucleate can have sizes comparable to isolated molecules or hexameric rings of RAD51 abundant in the nucleus, and are more likely to be suppressed by the noise removal algorithms described in Materials and Methods.

We observe that the fraction of RAD51 clusters adjacent to RPA (at C2C < 200 nm, see Supplementary Figure S2F) increases with time (Figure 4E) and peaks at 1.5 h to ∼70%. The distributions of distance between RPA and RAD51 structures (as estimated by the point-to-point distance P2P; Figure 4F) show that the proximity of RPA and RAD51 also increases to peak at 1.5 h, and stays constant thereafter. For those RAD51 filaments detected as having adjacent RPA clusters (C2C < 200 nm, see Supplementary Figure S8B and C), we observe that median inter-molecular P2P distances are <50 nm for all time points, reaching the lowest median values ∼25 ± 20 nm after 3 h (Figure 4F). Notably, the distance between BrdU-labeled ssDNA and RPA (see Figure 2E and F) clusters is much shorter (∼10 nm) than between RAD51 and RPA. Thus, whereas BrdU-labeled ssDNA and RPA co-localize at the molecular level, RPA and RAD51 adjoin closely without overlapping.

A different metric, the distance between the end of a RAD51 filament to the closest molecule contained in adjacent RPA cluster (see Supplementary Figure S8D and E, ‘end-to-point’ distance, or E2P, and Supplementary Figure S1H for definition of cluster ends) is even smaller (∼16 ± 13 nm). This provides further evidence that RPA clusters closely abut—but do not overlap—the ends of RAD51 filaments at a single DSB.

### BRCA2 is essential for initiation of RAD51 filaments at sites of DNA damage

To analyze the role of BRCA2, an essential mediator of RAD51 assembly, we first used siRNA directed against BRCA2 to deplete the encoded protein from cells. Consistent with prior work using confocal microscopy and *in vitro* studies, BRCA2 depletion inhibited the focalization of RAD51 clusters adjacent to RPA at sites of DNA damage and suppressed RAD51 filament extension as compared to control cells at matched time (Figure 5A–C and Supplementary Figure S12A and B), stars *** indicate a *P*-value—*P* < 0.001 obtained with two sample two-sided Wilcoxon rank sum test. However, d-STORM allowed us to assess the positional correlation between RAD51 and DSBs marked by RPA at a resolution well beyond conventional optical methods. In contrast to other time points, a relatively high fraction of RPA and RAD51 (∼40%) apparently co-localize at 30 min even after BRCA2 depletion (Figure 5D). However, the median P2P distance between RAD51 and RPA assemblies located within the radius of 200 nm is ∼90 nm (Figure 5G and Supplementary Figure S12C and D), in sharp contrast to control cells, where it is ∼25 nm (see Figure 4F). A similar conclusion is suggested by a decrease in the fraction of RAD51 co-localized with RPA (Figure 5D) to ∼10%, from ∼70% in control cells observed at later time points (Figure 4E). Together, these observations suggest that the apparent coincidence of RPA and RAD51 at 30 min after damage in BRCA2-depleted cells could arise from the random co-localization of small but widely distributed clusters. Therefore, to rigorously exclude artifacts due to random co-localization, we calculated the bivariate Ripley's K function (60) between centroids of RAD51 assemblies and centroids of RPA (Figure 5E, F, H and I), and compared it to the Ripley's function of randomized localizations of RAD51 (see Materials and Methods). This analysis confirms the strong co-localization between RAD51 and RPA after damage in the control cells, indicated by the experimental Ripley's function (violet) that lies far above the 95% confidence intervals (CI) of the randomized sample (blue) at 30 min (Figure 5F) and 5 h (Figure 5I). In contrast, however, there is no co-localization of RPA with RAD51 in BRCA2-depleted cells after DNA damage as exemplified by the analysis of Ripley's function at 30 min (Figure 5E) and 5 h (Figure 5H). Thus, these analyses confirm that the apparent coincidence of RAD51 and RPA clusters at 30 min after damage in BRCA2-depleted cells arises from random co-localization of independently distributed molecules. Furthermore, we observe even in undamaged cells (see Supplementary Figure S12I and J) RAD51 clusters of similar size and distribution to those detected 30 min after damage in BRCA2-depleted cells. This raises the possibility that such clusters may represent the BRCA2-independent accumulation of RAD51 at nuclear sites unconnected with IR-induced lesions (clearly visible in low resolution images, see Supplementary Figure S12H) or may correspond to nonspecific staining. Indeed, we observe that the median number of RAD51 molecules within a cluster is 10±4 at 30 min (Figure 5C) after BRCA2 depletion, below the value (18±7) observed in control cells (Figure 4B and Supplementary Figure S12B).

Interestingly, we find that BRCA2 depletion did not terminate end-resection, as reflected in the sustained accumulation of RPA after damage. The number of detected RPA molecules inside the cluster increases as compared to control cells (Figure 5J, Supplementary Figures S12E–G and S13) at each time point, except 5 h. This apparent pause between 3–5 h can be explained by random co-localization of RAD51 with RPA clusters of different size not always associated with IR-induced DNA damage. This observation suggests that in human cells DNA end-resection continues at damage sites even when RAD51 filament assembly does not proceed. Interestingly, the absence of a significant difference in RPA accumulation between BRCA2-depleted and matched control cells at 5 h raises the possibility that BRCA2 and RAD51 assembly proceeds independently from resection termination.

### BRC4 overexpression disrupts the assembly of RAD51 filaments

Overexpression of a peptide encoding the BRC4 repeat from human BRCA2, which mimics the RAD51 oligomerization interface, suppresses the formation of microscopic foci containing RAD51 at DNA damage sites (44,55). We therefore tested the effect of doxycycline-induced BRC4 expression on RPA and RAD51 localization using d-STORM microscopy (Supplementary Figure S14G and H). BRC4 expression not only eliminates RAD51 filament formation, but also prevents altogether RAD51 assembly at the sites of damage compared to control cells (Figure 6A–C, Supplementary Figure S14A), stars *** indicate a *P*-value—*P* < 0.001 obtained with two sample two-sided Wilcoxon rank sum test. Similar to BRCA2 depletion using siRNA, BRC4 expression abolishes RAD51 co-localization with RPA clusters (Figure 6D) and causes RAD51 dispersion resulting in large inter-assembly distances (Figure 6G, Supplementary Figure S14B–D). Ripley's function analyses (Figure 6E, F, H and I) confirmed that in cells over-expressing BRC4 peptide (Figure 6E and H), RAD51-RPA distribution is independent, in contrast to control cells (Figure 6F and I).

The median number of RAD51 molecules within a cluster is 13 ± 7 after BRC4 expression as compared to control cells 18 ± 9 at 30 min, (Figure 6C). Again, as with BRCA2 depletion using siRNA, BRC4 overexpression and impaired RAD51 filament assembly does not affect the progress of DNA end-resection marked by increased accumulation of RPA as compared to control cells (Figure 6J, Supplementary Figures S14E, F and S15).

### The C-terminal region of BRCA2 is essential for RAD51 filament extension but not filament initiation

Biochemical studies suggest that the RAD51-binding region at the C-terminus of BRCA2 stabilizes oligomers of RAD51 on DNA *in vitro* (48,49). To dissect the role of this region, we used a cell line, EUFA423, derived from a patient with Fanconi Anemia D1 complementation group (53). EUFA423 cells harbor compound heterozygosity for *BRCA2* mutations (*7691insAT* and *9900insA*) (78) that truncate the C-terminal region, but leave intact the region encoding the BRC repeats. We compared this cell line to a complemented derivative, EUFA423+BRCA2, stably expressing full-length BRCA2 (54,79). Strikingly, we observe that damage-induced RAD51 clusters are formed, but fail to elongate into extended filaments (Figure 7A and B), in *BRCA2* mutant EUFA423 cells. In contrast, RAD51 clusters progressively extend into filaments (Figure 7D and E) in BRCA2-complemented EUFA423 + BRCA2 cells (see Supplementary Figure S18 for comparison with LN9 fibroblast cell line obtained from clinically healthy individuals). Damage-induced focalization of RAD51 clusters adjacent to RPA appears to be unaffected by deletion of the C-terminal region of BRCA2. We observe that in EUFA423 + BRCA2 cells the median number of RAD51 molecules within a RAD51 assembly is 23 ± 13 at 30 min, as compared to EUFA423 cells having ∼29±22, then drops to ∼17 molecules at 1 h and stays constant (Figure 7B and E). The molecular content inside RAD51 filament is significantly different in EUFA423 compared to EUFA423+BRCA2 at any time, except at 30 min, stars *** indicate a *P*-value—*P* < 0.001 obtained with two sample two-sided Wilcoxon rank sum test. d-STORM images of DNA damage foci in EUFA423 and EUFA423+BRCA2 are shown with representative graphs (Figure 7K–N), plotting RAD51 in violet and RPA in green. We can still detect small RAD51 nuclei adjacent to RPA even in *BRCA2* mutant EUFA423 cells, and find no difference between EUFA423 and EUFA423+BRCA2 cells in the fraction of RAD51 and RPA structures that co-localize (Figure 7C and F). Similar conclusions arise from the distribution of inter-molecular distances between adjacent RAD51 and RPA clusters (P2P shown in Figure 7G and I; for all distances see Supplementary Figure S16C–F, I–L). We observe median values for RAD51 and RPA distance <50 nm in EUFA423 + BRCA2 (Figure 7I), which are even smaller in *BRCA2* mutant EUFA423, because in the absence of elongation of RAD51 assemblies, virtually all RAD51 detections occur in close proximity to RPA (Figure 7G).

EUFA423 cells express truncated forms of BRCA2 that contain the BRC repeat motifs (78). siRNA-mediated depletion of BRCA2 from these cells caused the delocalization of RAD51 from DSBs marked by RPA (Supplementary Figure S20), similar to the effects of BRCA2 depletion from HeLa cells (Figure 5). These findings confirm that the RAD51 clusters formed in EUFA423 cells are dependent on BRCA2, and further distinguish a requirement for the C-terminal region of BRCA2 in filament extension but not initiation.

Next, we tested whether the uncoupling between resection and RAD51 filament extension observed in HeLa Kyoto cells was affected by the C-terminal truncation of BRCA2. In EUFA423 + BRCA2 cells expressing full-length BRCA2, we observe a small, but gradual and consistent increase in the size of RPA clusters until 3 h after damage (Figure 7J, Supplementary Figures S16A and S17C, E) from which time it levels off, similar to HeLa Kyoto cells (Figure 4C). Remarkably, in *BRCA2* mutant EUFA423 cells, RPA extension continues even at later time points (Figure 7H and J, Supplementary Figures S16A, G and S17C–F), reaching larger extensions and molecular content at any given time when compared with EUFA423 + BRCA2 cells in which BRCA2 expression has been re-constituted.

One possible explanation for these larger RPA extensions is that end-resection continues unrestrained, without RAD51-RPA exchange, in *BRCA2* mutant EUFA423 cells. To test this possibility, we measured the total number of RAD51 and RPA detections for each co-localizing RAD51-RPA cluster (Supplementary Figure S16M). We observe that summed number of RPA and RAD51 molecules is higher in EUFA423 than EUFA423 + BRCA2 cells, suggesting a greater extend of resection unrestrained by RAD51 assembly. Together, these findings provide further evidence that DNA end-resection continues at damage sites even when RAD51 filament assembly does not proceed.

## DISCUSSION

**Figure 8.**
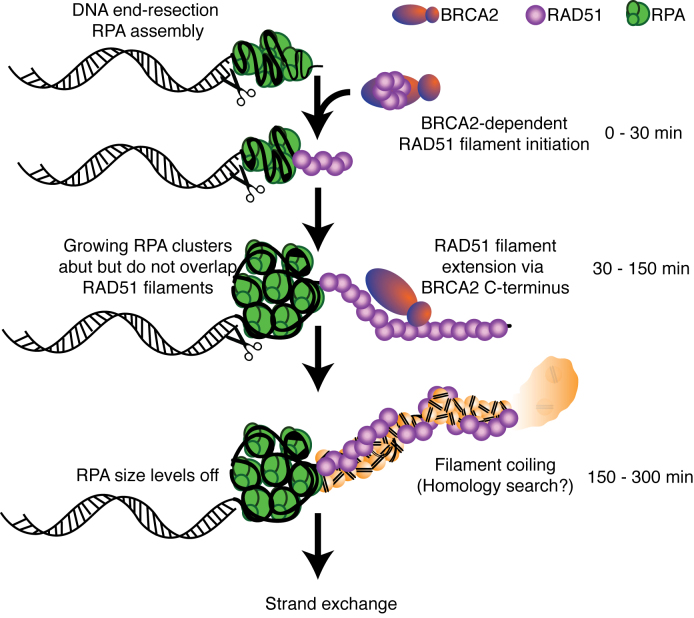
Schematic model of steps in DNA end-resection and RAD51 filament assembly during HR initiation at cellular DSBs. (0–30 min) Initiation of end-resection, RPA assembles on ssDNA. RAD51 assemblies abut RPA/ssDNA clusters, and requires the BRC repeats of BRCA2. (30–150 min) Continuing end-resection enlarges ssDNA and RPA clusters abutting RAD51. Elongation of RAD51 clusters into filaments is dependent on the C-terminal domain of BRCA2 (150–300 min). RPA cluster size levels off. RAD51 filaments assume a more compact and coiled structure, which could mark the homology search, leading eventually to strand exchange.

Our detailed analysis of RPA and RAD51 molecular assemblies at cellular sites of DSB repair carried out with super-resolving optical technologies at high spatial resolutions provides fresh insight into the mechanisms that control the spatiotemporal organization of RPA and RAD51 in their native cellular environment. We show that in wild-type HeLa epithelial cells, as well as human fibroblast EUFA423 cells complemented with BRCA2, the assembly of RAD51 filaments at DSB sites proceeds in three phases, namely: (i) the **initiation** of RAD51 filaments at around 20–30 min after damage, followed by (ii) **rapid elongation** at 1–1.5 h and (iii) a **slower elongation** phase from 3–5 h that is accompanied by apparent filament coiling (Figure 8). To define these phases, we have developed and deployed methods that involve efficient de-noising and filtering (Supplementary Figure S2G–H) of single-molecule microscopy data, as well as the quantification of structure size and co-localization metrics at the nanoscale level. Our methods detect very high—yet incomplete - co-localization fractions for markers that represent the same molecular intermediate (e.g. 90%, for RPA and BrdU labeling of ssDNA). More complete co-localization may be precluded in part by the limitations imposed by the efficiency of antibody labeling and co-detection. Moreover, molecules like RPA and RAD51 are abundant in the nucleoplasm as well as on chromatin. Therefore, efficient noise removal to exclude nucleoplasmic pools of isolated proteins may have the knock-on effect of compromising the detection of very small chromatin-bound clusters. We have carefully accounted for these factors when interpreting the results of our single-molecule localization studies.

Our findings reveal several interesting features of the molecular intermediates formed at cellular DSB sites during DNA end-resection and RAD51 filament assembly. Structural studies show (22,23,25,80) that RAD51 encases ssDNA in a one-start helix, in which each monomer engages ∼3 nt of ssDNA, with 6 RAD51 monomers per helical turn, and ∼9 nm helical pitch. We observe that typical length of RAD51 filaments at 3–5 h after damage is about 200 nm, which represents ∼400 bp of resected DNA covered by ∼ 130 RAD51 molecules. This length is in accord with estimates from the current literature (reviewed in (81)). We observe ∼80 (130) RAD51 molecules measured as median (mean) inside RAD51 filament at 3–5 h after damage. Therefore, we can detect at least ∼2 out of 3 RAD51 proteins. However, our resolution (∼40 nm) and sensitivity (∼5–10 molecules in the smallest clusters), may not be sufficient to detect initial nucleation of RAD51 filaments comprising possibly just one RAD51 dimer as suggested by *in vitro* studies using purified proteins (82,83). In addition, d-STORM, like other microscopy techniques involving antibody-labeling strategies, cannot provide absolute values of the protein content within an observed structure, and so the molecular contents reported in this manuscript should be understood as estimates. Nevertheless, d-STORM has much greater sensitivity than diffraction-limited microscopy, in which cluster of ∼5–10 proteins will be indistinguishable from the background, and so our work provides insights into the earliest molecular intermediates formed during RAD51 assembly in the native cellular environment.

RAD51 and RPA can be co-localized by conventional microscopy, but d-STORM has allowed us to distinguish that RPA localizes in large bundles adjacent but not interspersed with RAD51 clusters or filaments. The relative topography of RAD51 and RPA revealed by our studies raises the interesting possibility that RAD51 filament assembly occurs in a 3′ to 5′ direction on RPA-coated ssDNA strands. In other words, RPA may be displaced and RAD51 deposited as a 3′-ended strand of ssDNA is pulled out from an RPA-coated bundle (Figure 8). Intriguingly, a recent study using dSTORM [52] suggests that BRCA2 may be discretely positioned near the ends of extended RAD51 assemblies, consistent with BRCA2-mediated RAD51 deposition as we propose here.

Notably, we observed relatively linear RAD51 filaments adjacent to RPA-coated ssDNA bundles at early time points up to 1–1.5 h, that evolve over time into structures that frequently represent coiled RAD51 filament forms, at 3–5 h. This latter event may plausibly be explained in two ways. First, between 1.5 and 3 h, additional molecules may be recruited to elongating RAD51-ssDNA nucleoprotein filaments, causing changes in structure. Although a number of proteins might cause this, one candidate is RAD54. However, we detected no significant changes in RAD54 localization in this period. This raises a second possibility, namely that the transition in RAD51 filament conformation may conceivably mark homologous pairing with template duplex DNA, and the ensuing homology search. For instance, RAD54 forms regularly interspaced *puncta* along RAD51 filament, which may correspond to sites of interaction with target dsDNA. This provokes an interesting hypothesis, that steps in homology search may begin at an early stage during RAD51 filament assembly.

Our data define two functions for the tumor suppressor and HR mediator protein, BRCA2, in RAD51 filament assembly. First, as suggested by prior biochemical or cellular studies, the depletion of BRCA2, or overexpression of the RAD51-binding BRC4 repeat peptide, prevents RAD51 assembly at sites of DNA damage marked by RPA, and the subsequent formation of filaments. In addition, we posit a second function—for the C-terminal region of BRCA2—from studies on a cell line in which this region has been deleted. Strikingly, deletion of the C-terminal BRCA2 region allows RAD51 localization at sites of DNA damage and initial assembly of RAD51, but prevents the elongation of filaments. Such a defect, which may be compatible with limited HR execution, could explain the hypomorphic nature of bi-allelic mutations affecting the C-terminus of BRCA2 in patients with Fanconi anemia.

Interestingly, we present the first evidence in intact human cells that DNA end-resection, a prerequisite for HR initiation, occurs concurrently with RAD51 assembly at cellular sites of DSBs. First, RPA accumulation on BrdU-labeled ssDNA abuts but does not overlap with RAD51 clusters or filaments. In addition, RPA forms collapsed bundles rather than extended filaments like RAD51. Moreover, RPA accumulation on ssDNA progresses even when RAD51 clustering or elongation is inhibited, raising the possibility that end-resection may persist until a feedback signal marking successful homologous pairing is received. End-resection appears to occur independently from RAD51 loading on ssDNA although we cannot exclude that at later stages, when enough homology is detected to initiate DNA synthesis, a RAD51-dependent signal to stop resection exists. Finally, we observe a weak correlation, spearman correlation coefficient ∼0.1, between the number of detected molecules in RAD51 and RPA clusters (Supplementary Figure S9E). This, too, suggests that RAD51 loading is concurrent rather than consecutive with end-resection, in which case we would observe anti-correlation (*i.e*., large RPA clusters with short RAD51 filaments at early time-points, reversed at later time-points).

Collectively, our findings suggest a model for HR (Figure 8) wherein DNA end-resection and RPA loading on ssDNA are topographically uncoupled from RAD51 assembly, which occurs at abutting sites of ssDNA within a single radiation-induced repair focus. RAD51 accumulation and filament elongation leading to RPA displacement occur concurrently with resection and new RPA loading. The rarity of observations in which both RPA and RAD51 molecules are interspersed on a single filament suggests that human RAD51 may be loaded onto RPA-coated ssDNA complexes at a single site—or relatively few sites. However, because of limitations in the d-STORM technique to detect individual RAD51 nucleates, we cannot entirely exclude an interspaced pattern during very early nucleation (36).

Intriguingly, our work also defines kinetic changes in the localization of single molecules of RPA or RAD51 that may represent steps in HR. For instance, RAD51 filaments undergo a structural transition ∼3–5 h after damage, which we speculate may mark the onset of homologous pairing between presynaptic filaments, and target dsDNA that assumes a relaxed, coiled form.

In conclusion, our work exemplifies the power of quantitative super-resolution microscopy in dissecting a biochemical reaction in the native cellular milieu with unprecedented spatial resolution, and provides new insight into the mechanisms underlying a key biological process, DSB repair by RAD51-mediated HR.

## AVAILABILITY

All Matlab scripts used in this study are available in the Grafeo programme deposited in the GitHub repository: https://github.com/inatamara/Grafeo-dSTORM-analysis-

## Supplementary Material

Supplementary Data
