# Supplementary material for: Single-molecule localization microscopy reveals molecular transactions during RAD51 filament assembly at cellular DNA damage sites
Source: Nucleic Acids Res. 2018 Jan 4;46(5):2398–416. doi: 10.1093/nar/gkx1303 (PMC5861458; doi:10.1093/nar/gkx1303)
Supplement: Supplementary Data [file gkx1303_supp.pdf]

SUPPLEMENTARY DATA

Single-molecule localization microscopy reveals molecular transactions during RAD51 filament assembly at cellular DNA damage sites

Kalina T. Haas, MiYoung Lee, Alessandro Esposito and Ashok R. Venkitaraman

SUPPLEMENTARY FIGURE LEGENDS

Figure S1 (related to **Figure 1** and methods) Data filtering and clustering with Voronoi diagrams (VD) and Delaunay triangulation (DT) exemplified on synthetic data.

(A) Synthetic data were generated by adding detections randomly, modelled using Poisson homogenous (blue dots, background staining) and clustered (green dots, protein clusters, and red dots, centroids of a cluster) processes. **(B)** First, a Voronoi diagram is calculated (violet). Poisson homogenous processed are characterized by large Voronoi polygons (VP; yellow arrow) in contrast to clustered processes (red arrow and inset panel). **(C)** This allows for efficient data de-noising based on VP size distribution, by rejecting polygons with size smaller than a given threshold, typically the mean or median value of the observed distributions. **(D)** Next, Delaunay triangulation (DT) is computed. **(E)** The rejection of long DT edges allows us to segment protein clusters efficiently. **(F)** Compared to isolated points, clusters are characterised by small VP (blue) and short DT (red) edges. **(G)** Removing long DT edges results in discreet graphs, which **(H)** size can be estimated as maximum shortest path spanning the graph (red line connecting nodes 1 and 35, defined as 'ends' of the cluster). The unit-less scale bar length is the same for each figure.

Figure S2 (related to **Figure 1** and methods) Data filtering and clustering with Voronoi diagrams and Delaunay triangulation for two-colour d-STORM data.

Distributions of the localization precision of **(A)** Alexa647 and **(B)** CF568 fluorophores calculated according to Thompson's formalism (see main text). **(C)** The distribution of the number of photons emitted by single Alexa647 and CF568 molecules. **(D)** The distribution of the number of detections obtained from individual secondary antibodies spread on a glass coverslip. Prior to analysis with VP and DT we reject all single molecules with less than 1,000 photons detected **(C)** and with localization precision larger than 15 nm (**A,B**). **(E)** Next, corresponding VD are computed and thresholded around the mean of the VP density (inverse of VP size) distribution. Finally, isolated detections and small clusters are suppressed by thresholding bivariate **(F)** and univariate **(G)** inter-point distance distribution function from every point to 30 **(F)** or 10 **(G)** first nearest neighbours points of different **(F)** or the same **(G)** type at the distance < 150 nm (red vertical line).

Sampling density, the density of detected and analysed molecules, is approximated as the cubic root of the 3D VP **(H)** or square root of 2D VP (**I**). Sudden decrease in (**H**) corresponds to the threshold on the VP density shown in (**E**). Sampling density can be also approximated by the 1st nearest neighbour inter-point distance (**J**).

Figure S3 (related to **Figure 1** and methods) The analysis pipeline explained in Figures S1, S2 results in efficient de-noising and clustering of data, here exemplified by a representative input **(A)** and output **(B)** dataset pair. Correction of chromatic aberrations is performed using multicolour beads by applying wrap transformation (**C**). The shape of an image of a bead is elongated because chromatic aberration correction is performed on the same datasets used for the calibration of the z-position. In this case, the astigmatic lens is inserted and a z-sweep is performed.

Figure S4 (related to **Figure 2**) Structure of radiation-induced foci with BrdU-RPA generated using 5 Gy of X-rays radiation (IR).

(**A**) Number of detections in RPA clusters. Number of molecules, calculated by dividing number of detections by the expected number of detection from isolated antibody, inside RPA cluster represented on linear **(B)** and logarithmic scale **(C). (D)** Extension of RPA clusters. **(E)** Distribution of centroid-to-centroid distance (C2C) for every BrdU cluster to the closest RPA cluster. **(F)** Distribution of inter-point (point-to-point) distance (P2P) for every BrdU cluster to the closest RPA cluster. (**G**) Histogram of the centroid-to-centroid (C2C) distances between BrdU and RPA with distance threshold plot as red line. Abscissae indicate time after exposure to 5 Gy IR.

Figure S5 (related to **Figure 2**)

Exact p-values for the Max-t multiple mean comparison test for the number of molecules in (**A**) BrdU and (**C**) RPA cluster. Exact p-values for the Max-t multiple mean comparison test for the extension of (**B**) BrdU and (**D**) RPA cluster. (**E**) Spearman correlation coefficient between different measures of a size (number of molecules and extension) of BrdU and RPA clusters.

Figure S6 (related to **Figure 3**)

(A) Representative low resolution images of radiation-induced foci following DNA damage in HeLa Kyoto cells fixed from 5 min to 24 h after exposure to 5 Gy of IR and stained against RAD51 (magenta) and RPA (green). (**B**) Viability of HeLa Kyoto cells at 5 h and 24 h after exposure to 5 Gy of IR. Cells were trypsinized at indicated time points and viable cells were counted after staining with Trypan blue using Countess II (ThermoFisher). Mean values with SEM are shown. ns – not significant. (C) Number of RAD51 foci divided by area of the nucleus. Shortly, 3D stack images were acquired using Zeiss 880 Confocal Microscope using 40 X oil objective. Foci counting was performed on maximum intensity projection images using FindFoci Fiji plugin in batch processing

mode [1]. Cells with no RAD51 foci were counted as zero. Multiple group comparison was performed with Kruskal-Wallis H test; familywise error rate was corrected using Tukey-Kramer method. All groups were found different.

(D) Blow up DT super-resolved images of RAD51 (violet) and RPA (green). Red line serves as eye-guide of hypothetical RAD51 filament coiling. Scale bar – 250 nm.

Figure S7 (related to **Figure 3**) 2-dimensional projections of 3-dimensional d-STORM data. 3D d-STORM data can be represented as 2D scatter plot (**A**). 3D VP visualization for the z = 0 (**B**), y = 0 (**C**) or x = 0 (**D**). Scale bar – 250 nm.

Figure S8 (related to **Figure 4**) Structure of radiation-induced foci with RAD51-RPA staining.

(A) Extension of RPA clusters. **(B)** Distribution of inter-point distance (P2P) for every RAD51 cluster to the closest RPA cluster. **(C).** Distribution of C2C distance for RAD51 cluster having RPA cluster at C2C < 200 nm. **(D)** Distribution of end-to-point distance (E2P) for every RAD51 cluster to the closest RPA cluster. **(E)** Distribution of E2P distance for RAD51 cluster having RPA cluster at C2C < 200 nm. Abscissae indicate time after exposure to 5 Gy IR.

Figure S9 (related to **Figure 4**)

Exact p-values for the Max-t multiple mean comparison test for the extension of (**A**) RAD51 and (**D**) RPA cluster. Exact p-values for the Max-t multiple mean comparison test for the number of molecules in a RAD51 (**B**) and RPA cluster (**C**). (**E**) Spearman

correlation coefficient between different measures of a size (number of molecules and extension) of RAD51 and RPA clusters.

Figure S10 (related to **Figure 4**) Low-resolution and d-STORM images of radiation-induced foci following DNA damage in HPNE-hTERT human pancreatic cells. Scale bar – 250 nm.

(A) Representative low resolution images of HPNE cells fixed from 1 h and 3 h after exposure to 5 Gy of IR and stained against RAD51 (magenta) and RPA (green).

Representative d-STORM images of individual foci in HPNE cells fixed 1 h **(B)** and 3 h **(C)** after exposure to 5 Gy of IR and stained against RAD51 (magenta) and RPA (green). Scale bar – 250 nm.

Figure S11 (related to **Figure 4**) d-STORM images of radiation-induced foci of RAD51 (magenta) and RAD54 (green) following DNA damage in HeLa Kyoto cells.

Representative d-STORM images of RAD51 (magenta) and RAD54 (green) foci fixed 1.5 h (**A**) and 3 h (**C**) after exposure to 5 Gy of IR. Subset of images shown in A and C were used to plot cross-sectional intensities along RAD51 filament (red lines) at 1.5 h (**B**) and 3 h (**D**). The intensity profile at each point is given as sum of a 4-neighberhood (2x2) for each point on the line. Yellow stars indicate which filament profile was plotted. The Spearman correlation between the profile intensities of RAD51 and RAD54 distributions is shown as ρ. Black horizontal line indicates typical distance between two adjacent peaks of RAD54 (~100 nm). Pixel size, 10 nm. Scale bar, 250 nm.

Figure S12 (related to **Figure 5**) Structure of radiation-induced foci in HeLa cells after BRCA2 depletion characterised with RAD51-RPA staining**.**

RAD51 filament length (**A**) and number of molecules inside a filament (**B**) in match control cells for RAD51-RPA. (**C**) Distribution of centroid-to-centroid distance (C2C) for every RAD51 cluster to the closest RPA cluster. (**D**) Distribution of point-to-point distance (P2P) for every RAD51 cluster to the closest RPA cluster. (**E**) Extension of RPA clusters after siRNA mediated depletion of BRCA2. Number of molecules (**G**) and extension (**F**) of RPA cluster in matched control cells. (**H**) Representative low resolution images of BRCA2-depleted HeLa Kyoto cells fixed from 30 min and 3 h after exposure to 5 Gy of IR and stained against RAD51 (magenta) and RPA (green). (**I**) Representative image of non-irradiated Hela Kyoto cell stained against RAD51 (magenta) and RPA (green). (**J**) Close-ups of two nuclear regions showing RPA and RAD51 proximal localization in the absence of IR-induced DNA damage. Abscissae indicate time after exposure to 5 Gy IR.

Figure S13 (related to **Figure 5**)

Exact p-values for the Max-t multiple mean comparison test for the number of molecules (**A**) and extension (**B**) of RPA cluster in HeLa cells depleted with BRCA2.

Figure S14 (related to **Figure 6**) Structure of radiation induced foci in HeLa cells after BRC4 peptide expression characterised with RAD51-RPA staining.

(**A**) RAD51 filament length in match control cells for RAD51-RPA. (**B**) Distribution of point-to-point distance (P2P) for every RAD51 cluster to the closest RPA cluster. (**C**) Distribution of centroid-to-centroid C2C distance for every RAD51 cluster to the closest RPA cluster. (**D**) Distribution of C2C for RAD51 cluster having RPA cluster at C2C < 200 nm distance. (**E**) Extension of RPA clusters after BRC4 peptide overexpression. (**F**) Extension of RPA cluster in match control cells. Abscissae indicate time after exposure to 5 Gy IR. Representative low resolution images of HeLa Tet On clone 4.23 cells stably

expressing Myc-tagged BRC4 peptide fixed at 5 h after exposure to 5 Gy of IR and stained against Myc (red) and RPA (green) with doxycycline (1 µg/ml) **(G)** or without **(H)**.

Figure S15 (related to **Figure 6**)

Exact p-values for the Max-t multiple mean comparison test for the number of molecules (**A**) and extension (**B**) of RPA cluster in HeLa cells after BRC4 peptide expression.

Figure S16 (related to **Figure 7**) Structure of radiation induced foci in EUFA423+BRCA2 **(A-F)** and EUFA423 **(G-L)** cells characterised with RAD51-RPA staining.

(A) Extension of RPA clusters in EUFA423+BRCA2 cells. **(B)** Density of RAD51 assemblies. **(C)** Distribution of C2C distance for every RAD51 cluster to the closest RPA cluster. **(D)** Distribution of C2C distance for RAD51 cluster having RPA cluster at C2C < 200 nm. **(E)** Distribution of end-to-point distance (E2P) for every RAD51 cluster to the closest RPA cluster. **(F)** Distribution of end-to-point distance (E2P) for RAD51 cluster having RPA cluster at C2C < 200 nm.

(G) Extension of RPA clusters in EUFA423 cells. **(H)** Density of RAD51 assemblies. **(I)** Distribution of C2C distance for every RAD51 cluster to the closest RPA cluster. **(J)** Distribution of C2C distance for RAD51 cluster having RPA cluster at C2C < 200 nm. **(K)** Distribution of end-to-point distance (E2P) for every RAD51 cluster to the closest RPA cluster. **(L)** Distribution of end-to-point distance (E2P) for RAD51 cluster having RPA cluster at C2C < 200 nm.

(M) Total number of RAD51+RPA molecules indicating that resection length is comparable in EUFA423 and EUFA423 +BRCA2 cells and that lack of RPA replacement by RAD51 leads to increased RPA accumulation in EUFA423 cells. Abscissae indicate time after exposure to 5 Gy IR.

Figure S17 (related to **Figure 7**)

Exact p-values for the Max-t multiple mean comparison test for the extension of RAD51 filaments in EUFA423+BRCA2 (**A**) and EUFA423 (**B**) cells. Exact p-values for the Max-t multiple mean comparison test for number of molecules in RPA cluster in EUFA423+BRCA2 (**C**) and EUFA423 (**D**). Exact p-values for the Max-t multiple mean comparison test for the extension of RPA cluster in EUFA423+BRCA2 (**E**) and EUFA423 (**F**).

Figure S18 (related to **Figure 7**) Example images of radiation-induced foci following DNA damage in EUFA423, EUFA423+BRCA2 and LN9SV.

(**A**) Representative low resolution images of LN9SV cells, a control fibroblast obtained from clinically healthy individuals (left), the human BRCA2 deficient fibroblast EUFA423 (middle) and BRCA2-complemented fibroblasts EUFA423+BRCA2 (right). Cells were fixed from 3 h and 5 h after exposure to 5 Gy of IR and stained against RAD51 (magenta) and RPA (green). Representative DT visualizations of RAD51 (violet) and RPA (green) molecules at radiation-induced foci in LN9SV cells at 3 h (**B**) and 5 h (**C**) after exposure to 5 Gy IR. Scale bar – 250 nm.

Figure S19 (related to **Figure 7**) Viability of EUFA423 (**A**) and EUFA423+BRCA2 (**B**) after exposure to 5 Gy IR.

Each cell line was plated in 6 well plates in triplicate. Cells were trypsinized at indicated time points and viable cells were counted after staining with Trypan blue using Countess II (ThermoFisher). Mean values with SEM are shown. * $p<0.05$, ($p=0.0478$)

Figure S20 (related to **Figure 7**) Topography of RAD51 and RPA at radiation-induced foci in EUFA423 cells after siRNA-induced depletion of BRCA2.

(**A**) Distribution of the number of RAD51 molecules inside RAD51 cluster in EUFA423+siBRCA2 (siB2) and mock transfected EUFA423 cells. (**B**) Distribution of the number of molecules inside RPA cluster in EUFA423+siBRCA2 (siB2) and mock transfected EUFA423 cells. In siRNA BRCA2 number of molecules in RPA cluster saturates faster than in control cells, which can be explained by random RAD51 location in the proximity of small and large RPA clusters. (**C**) Bivariate Ripley's function for RAD51 and RPA in EUFA423 + siRNA BRCA2 (right) and mock transfected control cells (left) for 5 h after exposure to 5 Gy IR. The purple line marks the experimental Ripley's function while the shaded blue areas and the blue lines show the 95% confidence intervals (CI) and mean obtained by repetitive randomization of cluster centroid positions. (**D**) RAD51 (purple) and RPA (green) foci represented as DT in EUFA423 (left) and EUFA423 + siBRCA2 (right). Threshold for conditional bivariate and univariate distance distribution function was set as described in Material & Methods cluster analysis subsection. Distributions were computed over data aggregated from two replicates. Abscissae indicate time after exposure to 5 Gy IR. Scale bar – 250 nm.

1. Herbert, A.D., A.M. Carr, and E. Hoffmann, *FindFoci: A Focus Detection Algorithm with Automated Parameter Training That Closely Matches Human Assignments, Reduces Human Inconsistencies and Increases Speed of Analysis.* PLoS ONE, 2014. **9**(12): p. e114749.

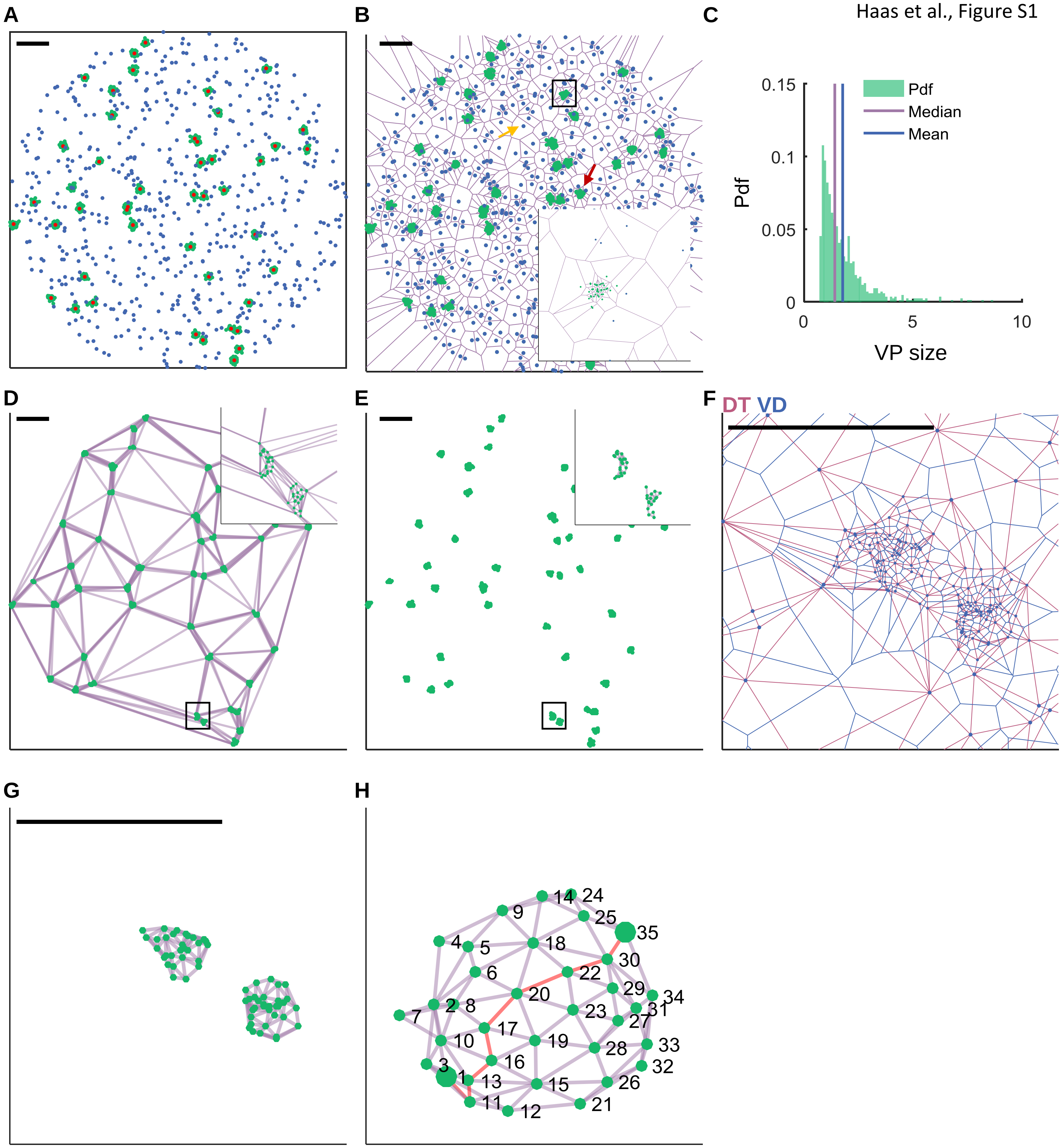
A
B
C
Pdf
Median
Mean
0.15
0.1
0.05
0
5
10
VP size
D
E
F
DT VD
G
H
1
2
3
4
5
6
7
8
9
10
11
12
13
14
15
16
17
18
19
20
21
22
23
24
25
26
27
28
29
30
31
32
33
34
35

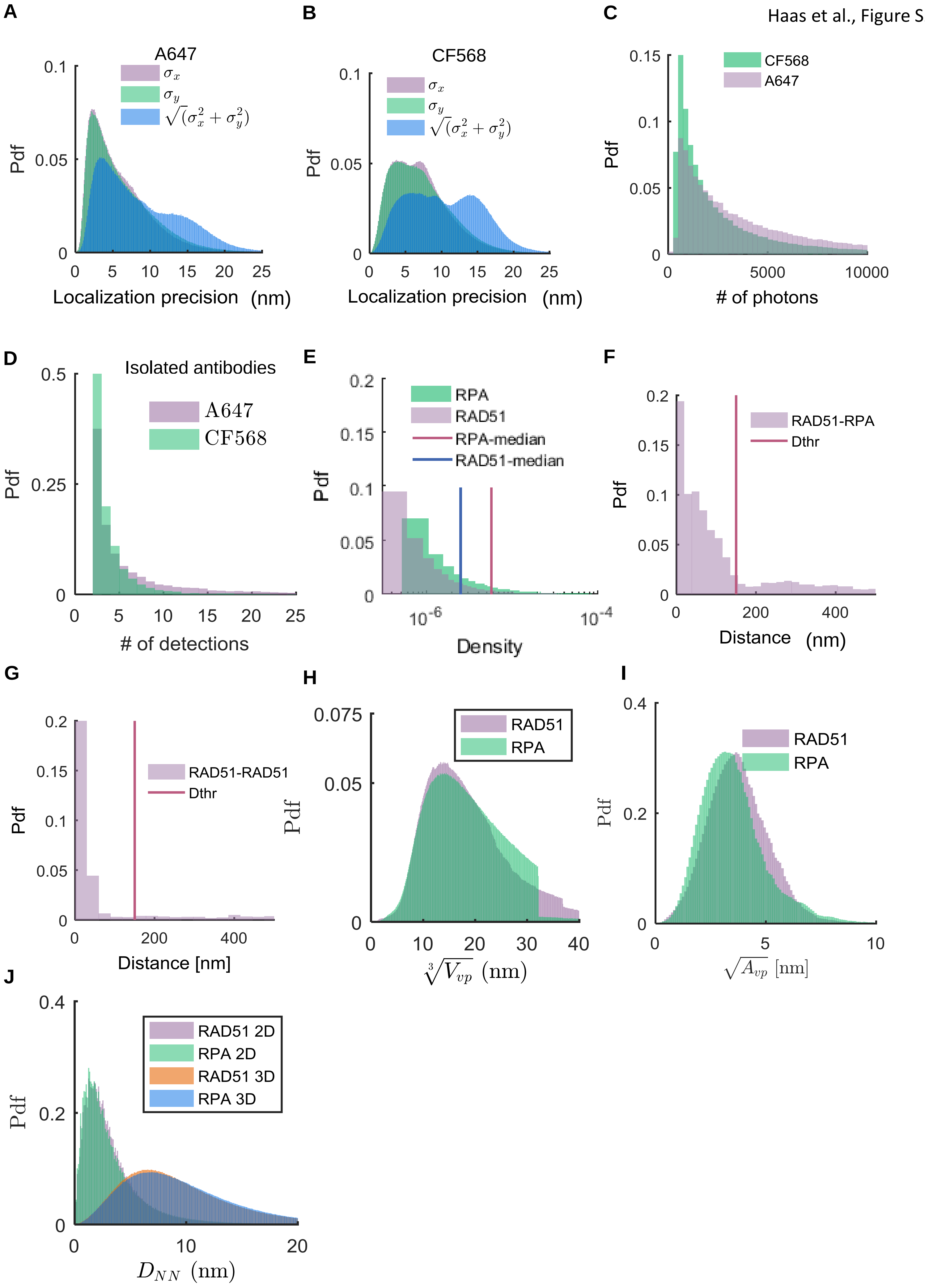
A
A647
Pdf
0.1
0.05
0
0
5
10
15
20
25
Localization precision (nm)
B
CF568
0.1
0.05
0
Localization precision (nm)
C
CF568
A647
0.15
0.1
0.05
0
5000
10000
# of photons
D
Isolated antibodies
A647
CF568
0.5
0.25
# of detections
E
RPA
RAD51
RPA-median
RAD51-median
0.2
0.15
Density
F
RAD51-RPA
Dthr
200
400
Distance (nm)
G
RAD51-RAD51
Dthr
Distance [nm]
H
RAD51
RPA
0.075
I
0.4
0.2
J
RAD51 2D
RPA 2D
RAD51 3D
RPA 3D

A

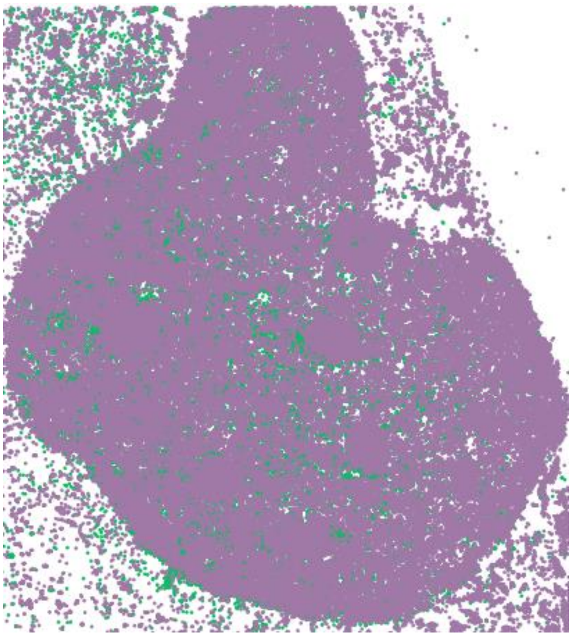

B

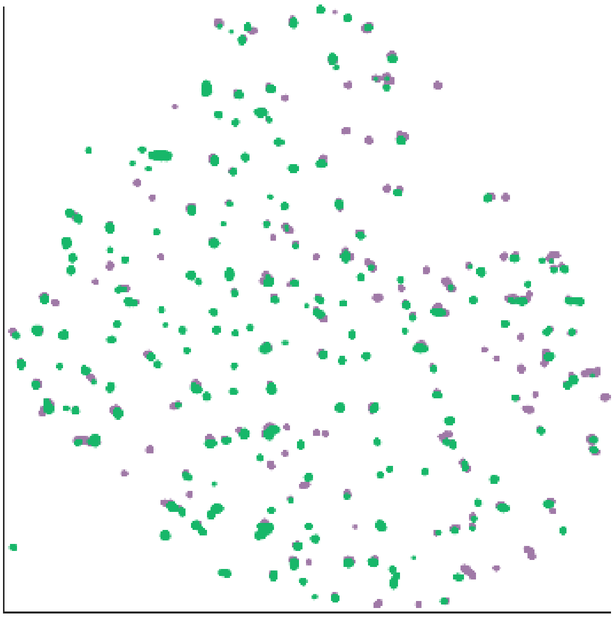

C

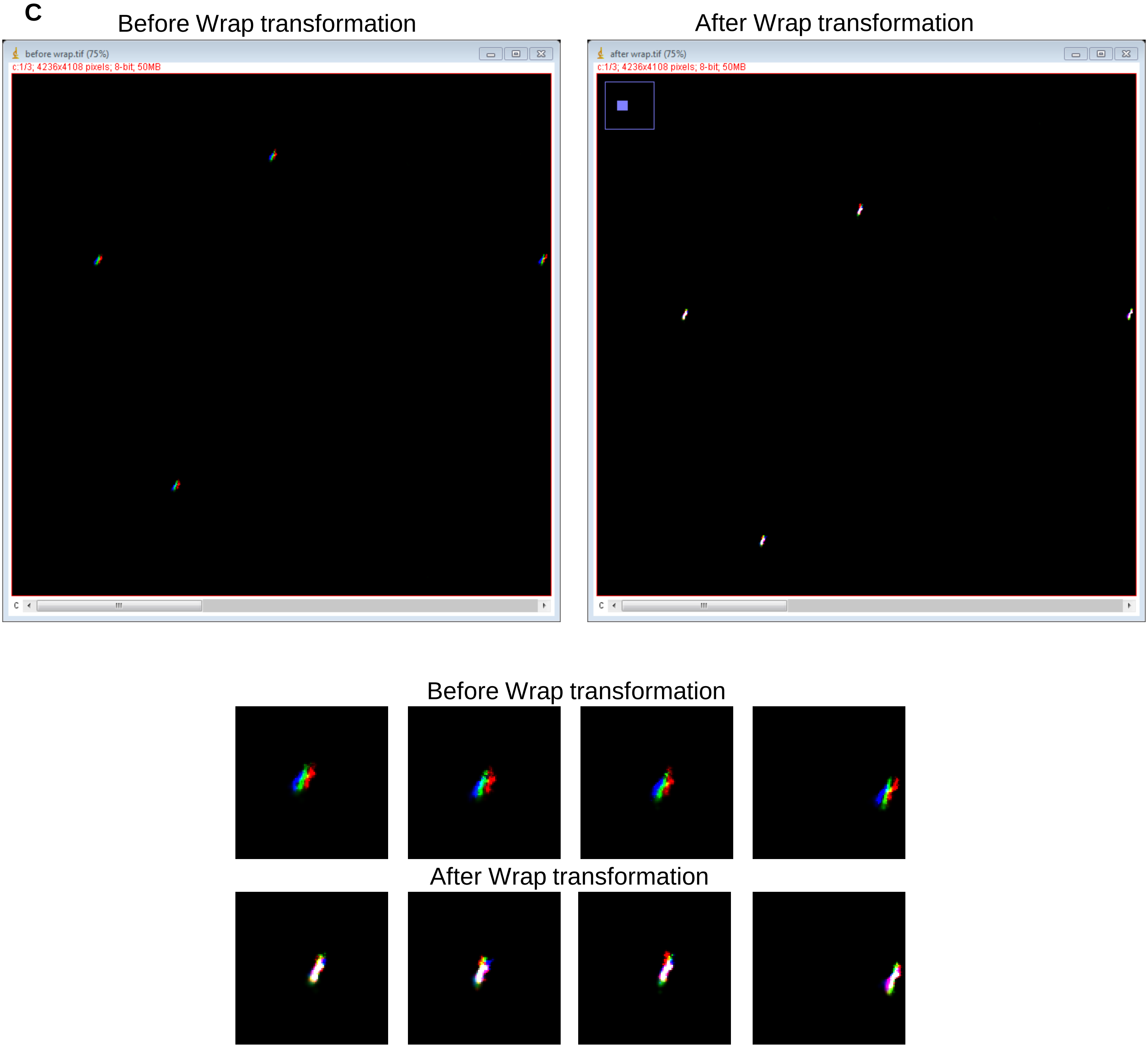
Before Wrap transformation
After Wrap transformation
before wrap.tif (75%)
c:1/3; 4236x4108 pixels; 8-bit; 50MB
after wrap.tif (75%)
c:1/3; 4236x4108 pixels; 8-bit; 50MB
Before Wrap transformation
After Wrap transformation

BrdU-RPA

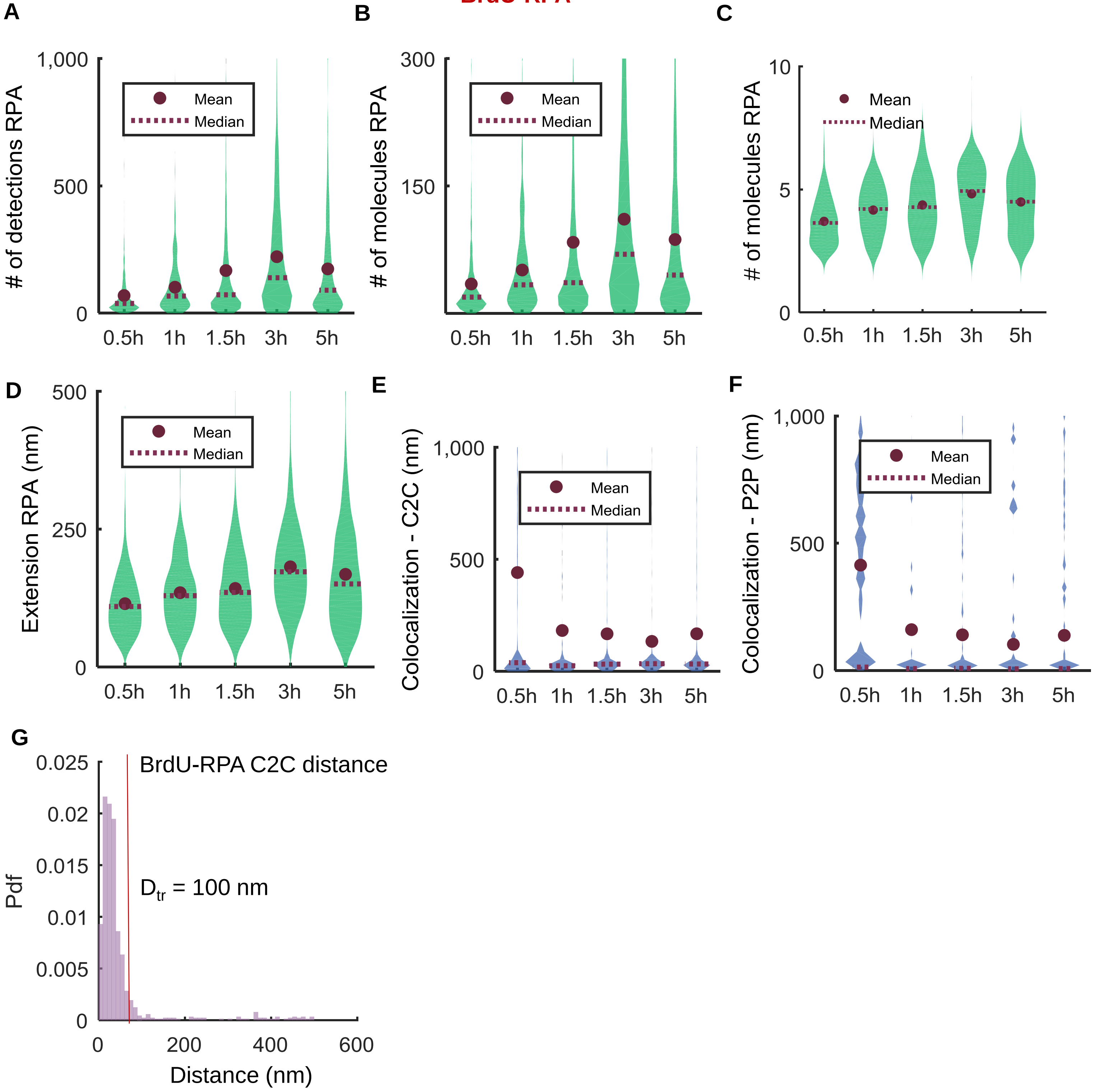
A
# of detections RPA
1,000
500
0
Mean
Median
0.5h
1h
1.5h
3h
5h
B
# of molecules RPA
300
150
C
# of molecules RPA
10
5
D
Extension RPA (nm)
500
250
E
Colocalization - C2C (nm)
1,000
500
F
Colocalization - P2P (nm)
G
Pdf
0.025
0.02
0.015
0.01
0.005
0
BrdU-RPA C2C distance
D_{tr} = 100 nm
0
200
400
600
Distance (nm)

Null Hypotheses	Ptukey $\alpha = 0.05$
0.5h = 1h	4.8×10^{-11}
0.5h = 1.5h	2.4×10^{-10}
0.5h = 3h	0
0.5h = 5h	0
1h = 1.5h	0.29
1h = 3h	1.2×10^{-4}
1h = 5h	2.4×10^{-14}
1.5h = 3h	1.7×10^{-12}
1.5h = 5h	0
3h = 5h	0.01

Supplementary table A
BrdU no. of molecules
Max-t test for multiple mean comparison

Null Hypotheses	Ptukey $\alpha = 0.05$
0.5h = 1h	7.4×10^{-13}
0.5h = 1.5h	0
0.5h = 3h	0
0.5h = 5h	0
1h = 1.5h	0.013
1h = 3h	0
1h = 5h	7.4×10^{-13}
1.5h = 3h	5.7×10^{-13}
1.5h = 5h	0.17
3h = 5h	3×10^{-6}

Supplementary table C
RPA no. of molecules
Max-t test for multiple mean comparison

Null Hypotheses	Ptukey $\alpha = 0.05$
0.5h = 1h	1.2×10^{-13}
0.5h = 1.5h	0
0.5h = 3h	0
0.5h = 5h	0
1h = 1.5h	0.91
1h = 3h	1.6×10^{-6}
1h = 5h	7.3×10^{-4}
1.5h = 3h	4.8×10^{-13}
1.5h = 5h	2.3×10^{-8}
3h = 5h	0.54

Supplementary table B
BrdU Extension
Max-t test for multiple mean comparison

Null Hypotheses	Ptukey $\alpha = 0.05$
0.5h = 1h	3.3×10^{-7}
0.5h = 1.5h	0
0.5h = 3h	0
0.5h = 5h	0
1h = 1.5h	0.50
1h = 3h	0
1h = 5h	1.7×10^{-5}
1.5h = 3h	0
1.5h = 5h	2.2×10^{-4}
3h = 5h	3.8×10^{-8}

Supplementary table D
RPA Extension
Max-t test for multiple mean comparison

Var1-Var2	Time				
	0.5h	1h	1.5h	3h	5h
Mol.BrdU - Mol.RPA	0.42	0.72	0.47	0.46	0.72
Ext.BrdU - Ext.RPA	0.55	0.75	0.64	0.69	0.75
Mol.BrdU - Ext.BrdU	0.82	0.82	0.86	0.86	0.88
Mol.RPA - Ext.RPA	0.73	0.86	0.72	0.80	0.88

Supplementary table E
HeLa Kyoto, Spearman rank correlation coefficient
for RPA and BrdU cluster measures

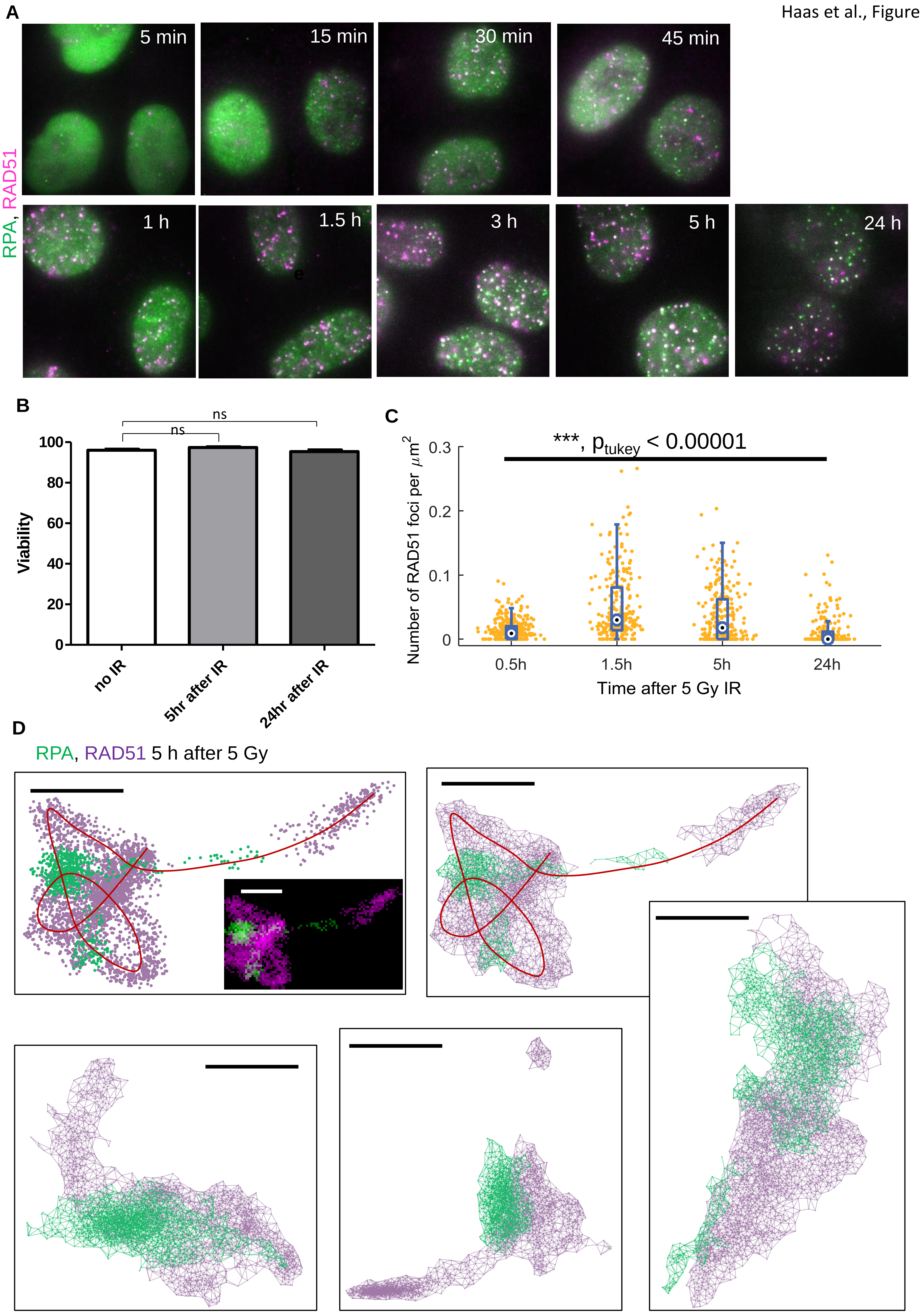
A
5 min
15 min
30 min
45 min
1 h
1.5 h
3 h
5 h
24 h
RPA, RAD51
B
ns
ns
Viability
100
80
60
40
20
0
no IR
5hr after IR
24hr after IR
C
***, p_{tukey} < 0.00001
Number of RAD51 foci per μm^2
0.3
0.2
0.1
0
0.5h
1.5h
5h
24h
Time after 5 Gy IR
D
RPA, RAD51 5 h after 5 Gy

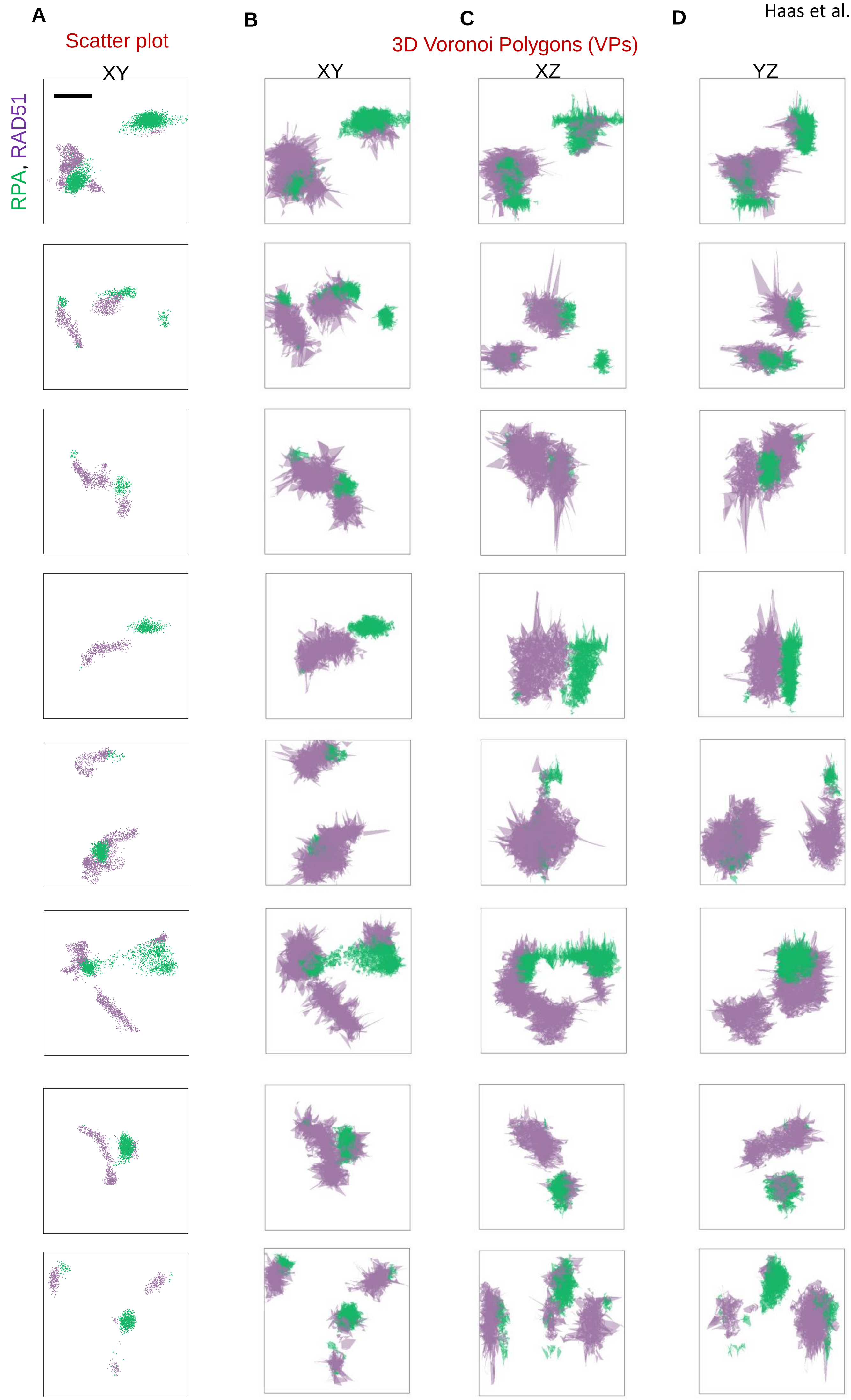
A
B
C
D
Scatter plot
3D Voronoi Polygons (VPs)
XY
XY
XZ
YZ
RPA, RAD51

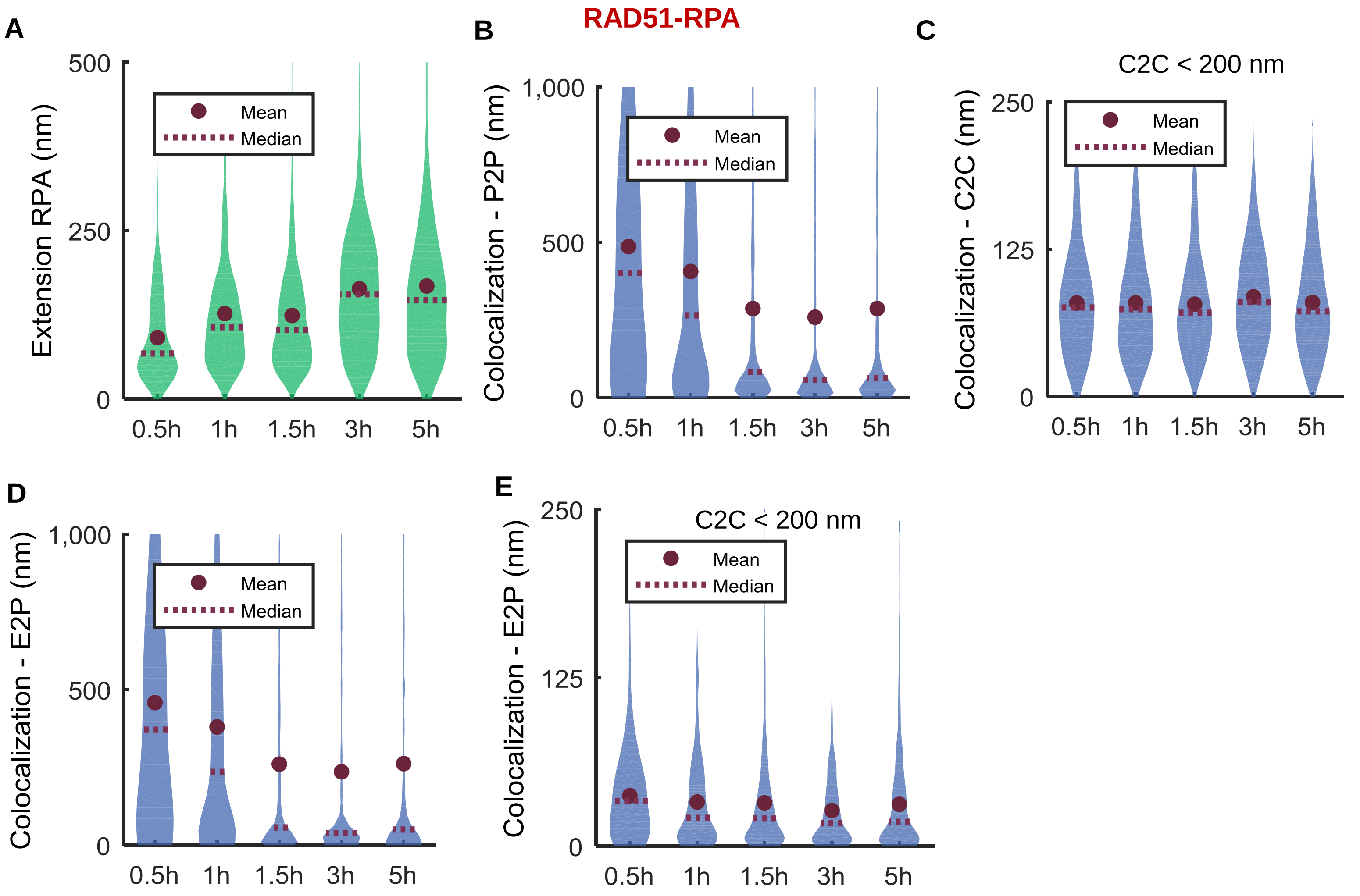
RAD51-RPA
A
Extension RPA (nm)
500
250
0
Mean
Median
0.5h
1h
1.5h
3h
5h
B
Colocalization - P2P (nm)
1,000
500
0
Mean
Median
0.5h
1h
1.5h
3h
5h
C
C2C < 200 nm
Colocalization - C2C (nm)
250
125
0
Mean
Median
0.5h
1h
1.5h
3h
5h
D
Colocalization - E2P (nm)
1,000
500
0
Mean
Median
0.5h
1h
1.5h
3h
5h
E
C2C < 200 nm
Colocalization - E2P (nm)
250
125
0
Mean
Median
0.5h
1h
1.5h
3h
5h

**Null Hypotheses**	**Ptukey** $\alpha = 0.05$
0.5h = 1h	7.2×10^{-6}
0.5h = 1.5h	0
0.5h = 3h	0
0.5h = 5h	0
1h = 1.5h	0
1h = 3h	0
1h = 5h	0
1.5h = 3h	3×10^{-13}
1.5h = 5h	0
3h = 5h	0.52

Supplementary table A
HeLa Kyoto, RAD51 Extension
Max-t test for multiple mean comparison

**Null Hypotheses**	**Ptukey** $\alpha = 0.05$
0.5h = 1h	0
0.5h = 1.5h	0
0.5h = 3h	0
0.5h = 5h	0
1h = 1.5h	0.99
1h = 3h	0
1h = 5h	0
1.5h = 3h	0
1.5h = 5h	0
3h = 5h	0.31

Supplementary table C
HeLa Kyoto, RPA no. of molecules
Max-t test for multiple mean comparison

**Null Hypotheses**	**Ptukey** $\alpha = 0.05$
0.5h = 1h	0
0.5h = 1.5h	1.2×10^{-16}
0.5h = 3h	0
0.5h = 5h	0
1h = 1.5h	0
1h = 3h	0
1h = 5h	2×10^{-3}
1.5h = 3h	0
1.5h = 5h	0
3h = 5h	6×10^{-7}

Supplementary table B
HeLa Kyoto, RAD51 no. of molecules
Max-t test for multiple mean comparison

**Null Hypotheses**	**Ptukey** $\alpha = 0.05$
0.5h = 1h	1×10^{-12}
0.5h = 1.5h	1×10^{-12}
0.5h = 3h	0
0.5h = 5h	0
1h = 1.5h	0.73
1h = 3h	0
1h = 5h	6×10^{-15}
1.5h = 3h	0
1.5h = 5h	0
3h = 5h	0.89

Supplementary table D
HeLa Kyoto, RPA Extension
Max-t test for multiple mean comparison

**Var1-Var2**	**Time**				
	0.5h	1h	1.5h	3h	5h
Mol.RAD51 - Mol.RPA	0.12	0.10	0.06	0.11	0.16
Ext.RAD51 - Ext.RPA	0.15	0.17	0.07	0.14	0.22
Mol.RAD51 - Ext.RAD51	0.77	0.86	0.85	0.9	0.86
Mol.RPA - Ext.RPA	0.89	0.89	0.88	0.90	0.86
Ext.RPA - E2B	-0.14	-0.15	-0.25	-0.26	-0.28

Supplementary table E
HeLa Kyoto, Spearman rank correlation coefficient
for RAD51 and RPA cluster measures

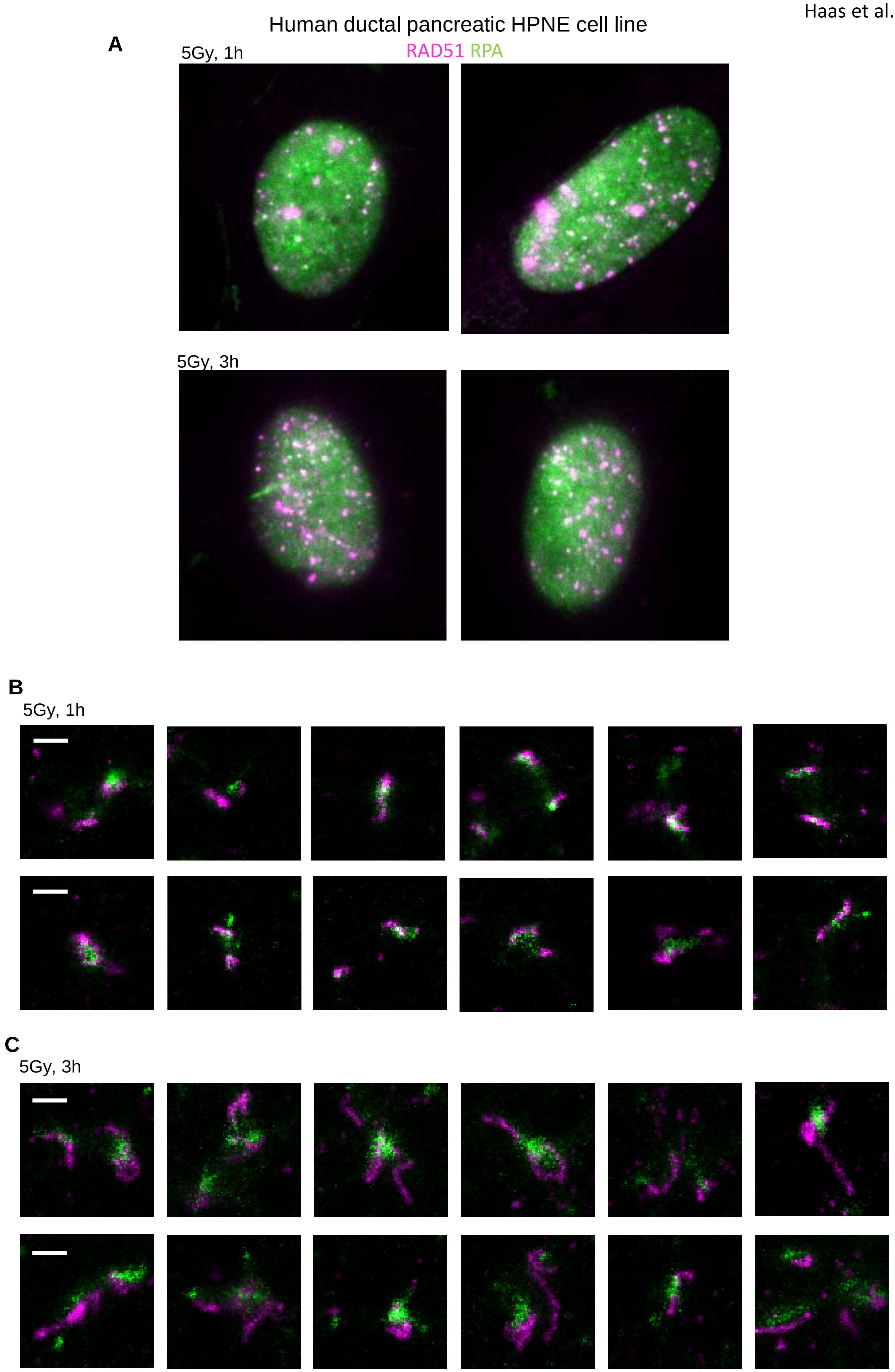
Human ductal pancreatic HPNE cell line
A
5Gy, 1h
RAD51 RPA
5Gy, 3h
B
5Gy, 1h
C
5Gy, 3h

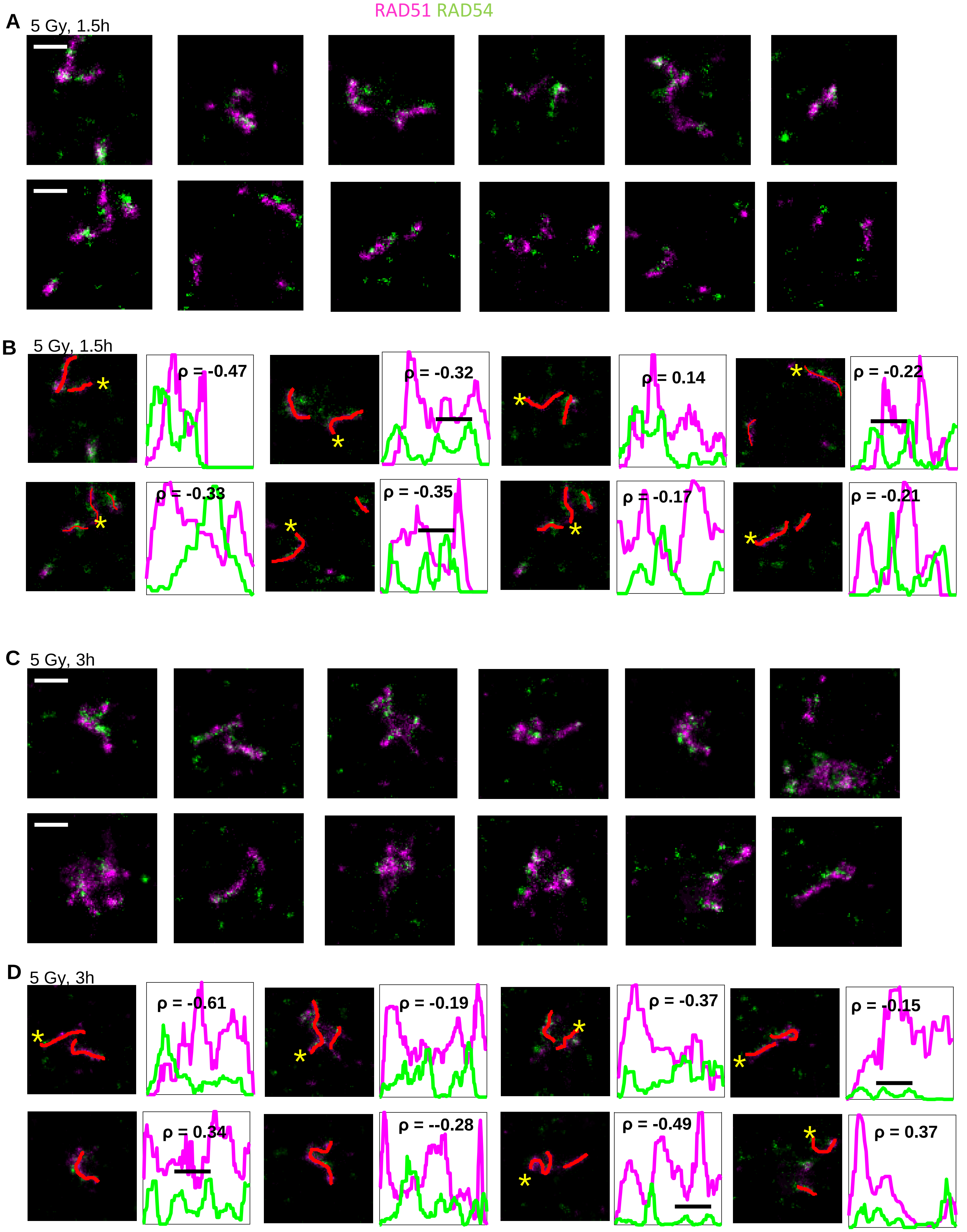
RAD51 RAD54
A
5 Gy, 1.5h
B
5 Gy, 1.5h
ρ = -0.47
ρ = -0.32
ρ = 0.14
ρ = -0.22
ρ = -0.33
ρ = -0.35
ρ = -0.17
ρ = -0.21
C
5 Gy, 3h
D
5 Gy, 3h
ρ = -0.61
ρ = -0.19
ρ = -0.37
ρ = -0.15
ρ = 0.34
ρ = --0.28
ρ = -0.49
ρ = 0.37

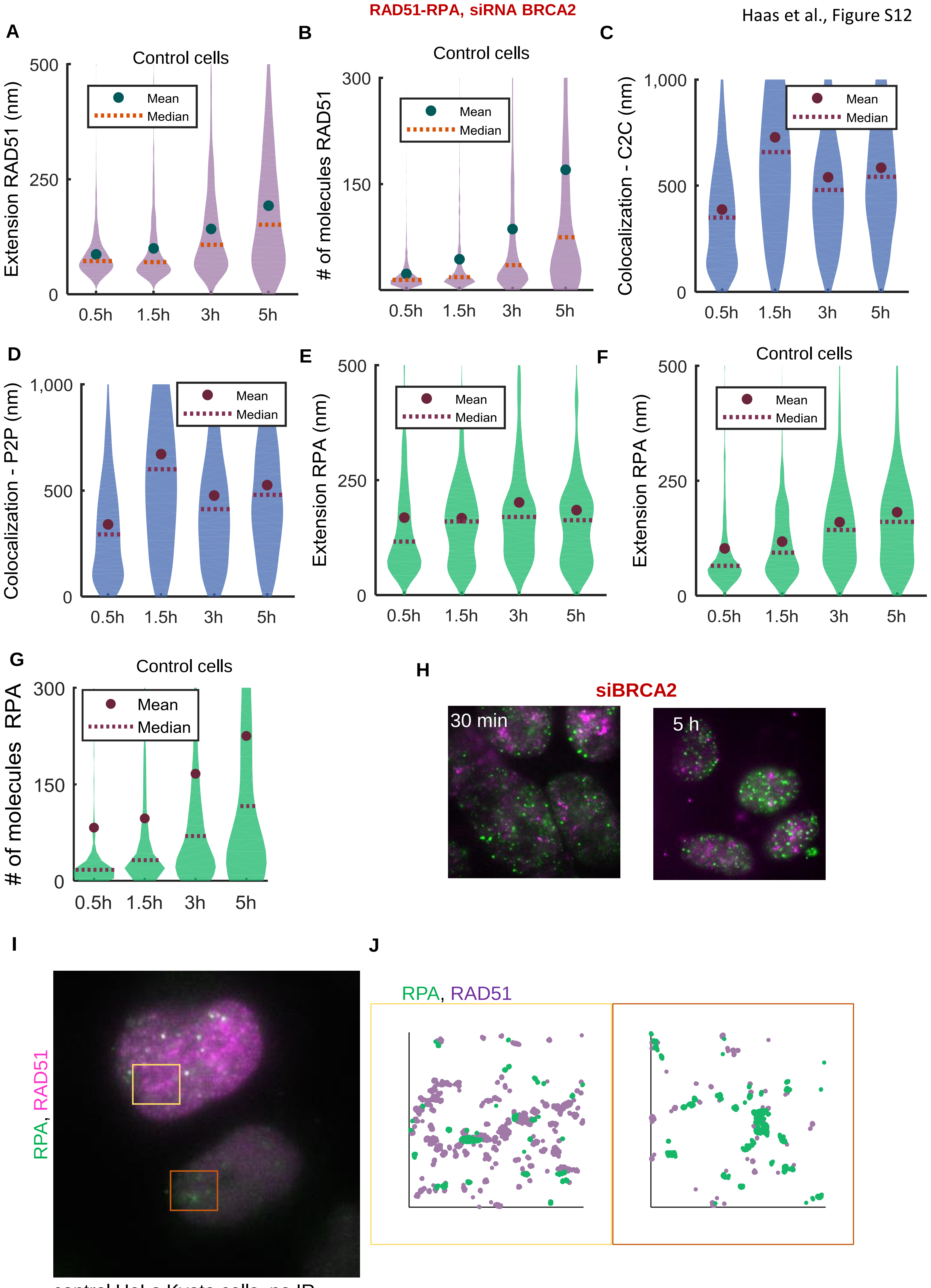

control HeLa Kyoto cells, no IR

Null Hypotheses	Ptukey $\alpha = 0.05$
0.5h = 1.5h	4×10^{-4}
0.5h = 3h	0
0.5h = 5h	2×10^{-7}
1.5h = 3h	3×10^{-10}
1.5h = 5h	0.6
3h = 5h	8×10^{-8}

Supplementary table A
HeLa Kyoto siBRCA2, RPA no. of molecules
Max-t test for multiple mean comparison

Null Hypotheses	Ptukey $\alpha = 0.05$
0.5h = 1.5h	0
0.5h = 3h	0
0.5h = 5h	0
1.5h = 3h	3.9×10^{-2}
1.5h = 5h	4.9×10^{-5}
3h = 5h	0.25

Supplementary table B
HeLa Kyoto siBRCA2, RPA Extension
Max-t test for multiple mean comparison

RAD51-RPA, oeBRC4

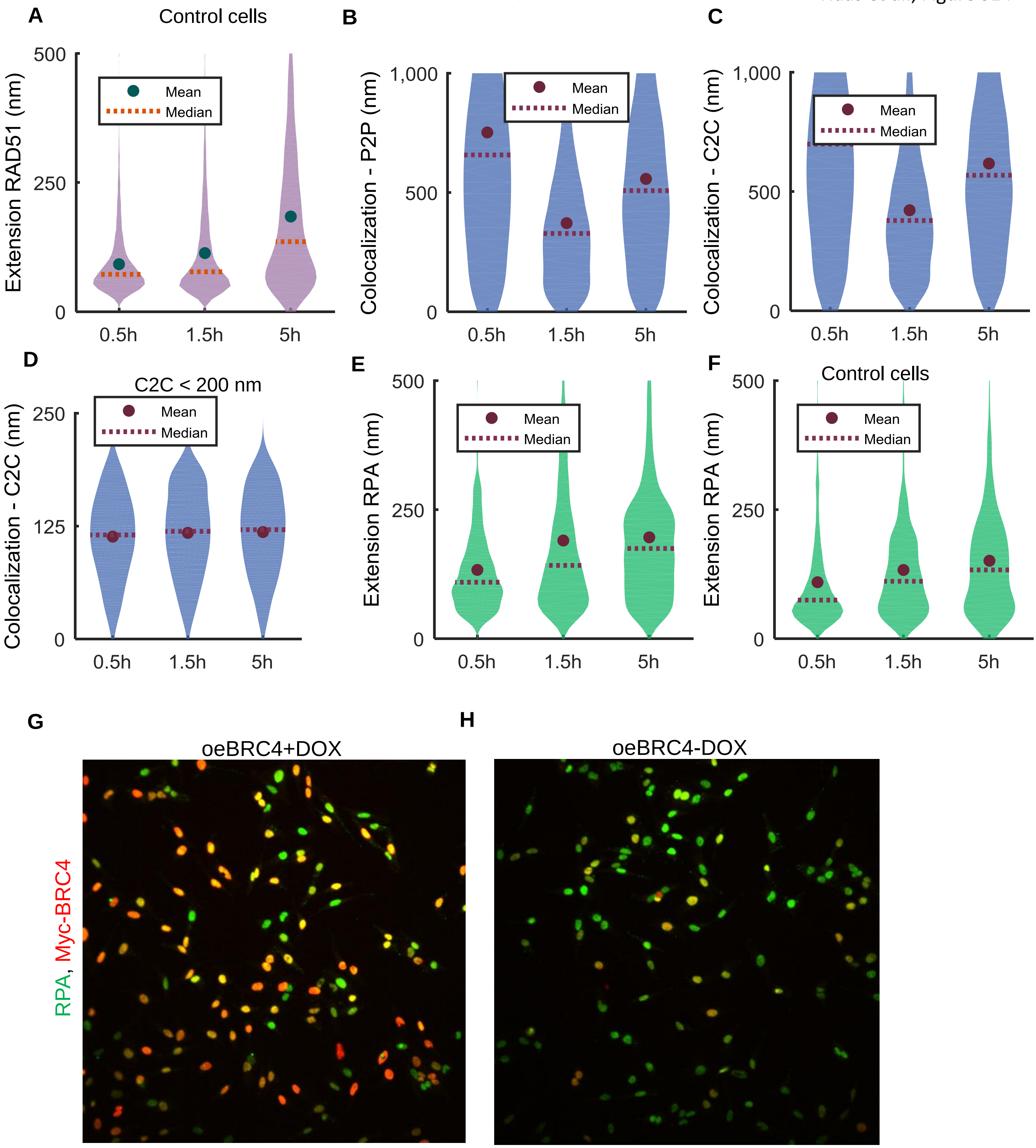

Null Hypotheses	Ptukey $\alpha = 0.05$
0.5h = 1.5h	1.4×10^{-14}
0.5h = 5h	0
1.5h = 5h	0

Supplementary table A
HeLa Kyoto oeBRC4, RPA no. of molecules
Max-t test for multiple mean comparison

Null Hypotheses	Ptukey $\alpha = 0.05$
0.5h = 1.5h	0
0.5h = 5h	0
1.5h = 5h	0

Supplementary table B
HeLa Kyoto oeBRC4, RPA Extension
Max-t test for multiple mean comparison

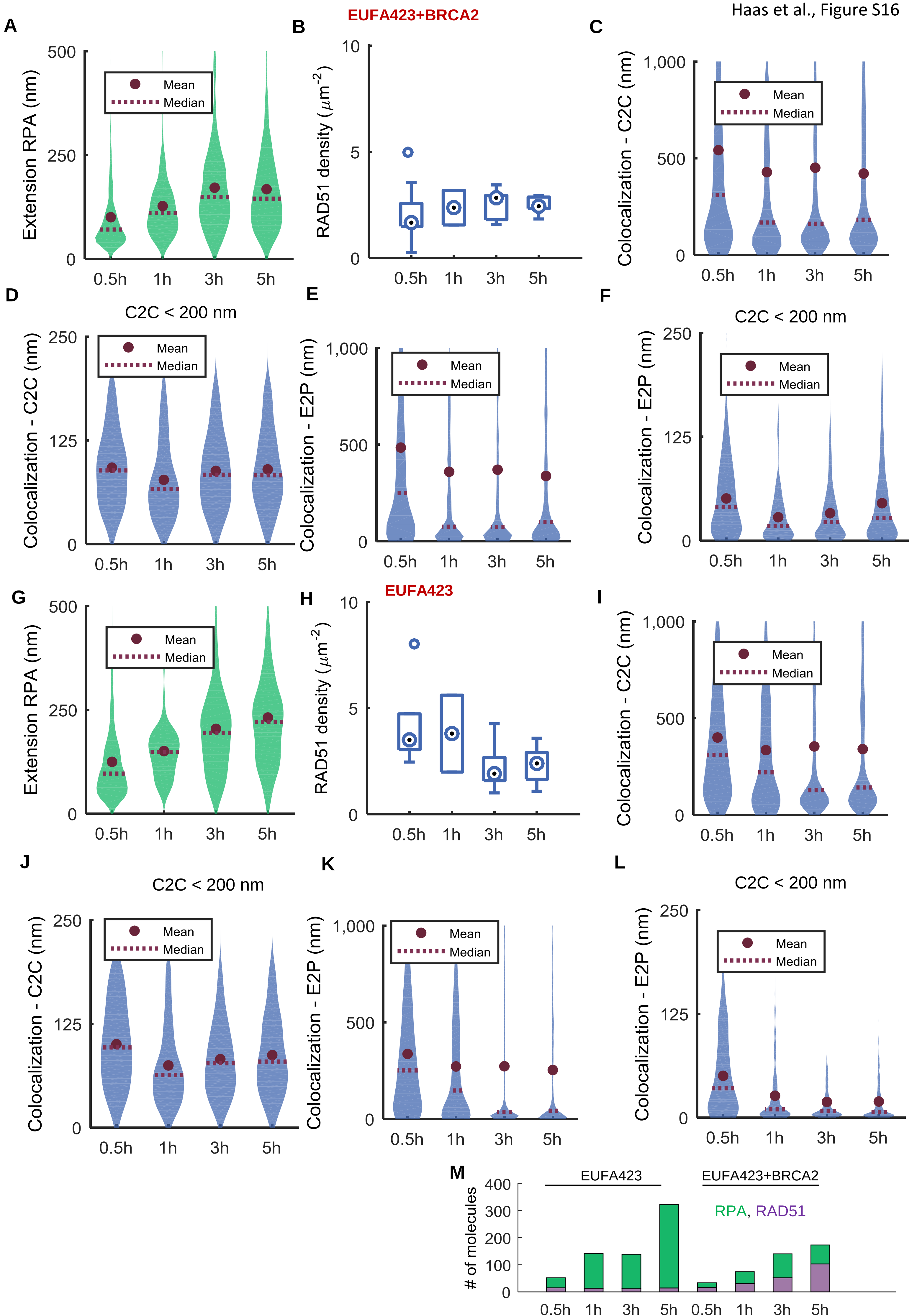
EUFA423+BRCA2
A
B
C
D
E
F
G
H
I
J
K
L
M
Extension RPA (nm)
RAD51 density (μm^{-2})
Colocalization - C2C (nm)
Colocalization - E2P (nm)
C2C < 200 nm
Mean
Median
0.5h
1h
3h
5h
EUFA423
EUFA423+BRCA2
RPA, RAD51
# of molecules

Null Hypotheses	Ptukey $\alpha = 0.05$
0.5h = 1h	0
0.5h = 3h	0
0.5h = 5h	0
1h = 3h	0.22
1h = 5h	0
3h = 5h	0

Supplementary table A
EUFA423+BRCA2, RAD51 Extension
Max-t test for multiple mean comparison

Null Hypotheses	Ptukey $\alpha = 0.05$
0.5h = 1h	0.31
0.5h = 3h	0.007
0.5h = 5h	1.2×10^{-8}
1h = 3h	1.4×10^{-4}
1h = 5h	7×10^{-10}
3h = 5h	0.005

Supplementary table B
EUFA423, RAD51 Extension
Max-t test for multiple mean comparison

Null Hypotheses	Ptukey $\alpha = 0.05$
0.5h = 1h	0
0.5h = 3h	0
0.5h = 5h	0
1h = 3h	0.007
1h = 5h	0.015
3h = 5h	0.98

Supplementary table C
EUFA423+BRCA2, RPA no. of molecules
Max-t test for multiple mean comparison

Null Hypotheses	Ptukey $\alpha = 0.05$
0.5h = 1h	0
0.5h = 3h	0
0.5h = 5h	0
1h = 3h	0.14
1h = 5h	2×10^{-13}
3h = 5h	0

Supplementary table D
EUFA423, RPA no. of molecules
Max-t test for multiple mean comparison

Null Hypotheses	Ptukey $\alpha = 0.05$
0.5h = 1h	0
0.5h = 3h	0
0.5h = 5h	0
1h = 3h	3×10^{-15}
1h = 5h	2×10^{-13}
3h = 5h	0.84

Supplementary table E
EUFA423+BRCA2, RPA Extension
Max-t test for multiple mean comparison

Null Hypotheses	Ptukey $\alpha = 0.05$
0.5h = 1h	0
0.5h = 3h	0
0.5h = 5h	0
1h = 3h	0
1h = 5h	1×10^{-6}
3h = 5h	0

Supplementary table F
EUFA423, RPA Extension
Max-t test for multiple mean comparison

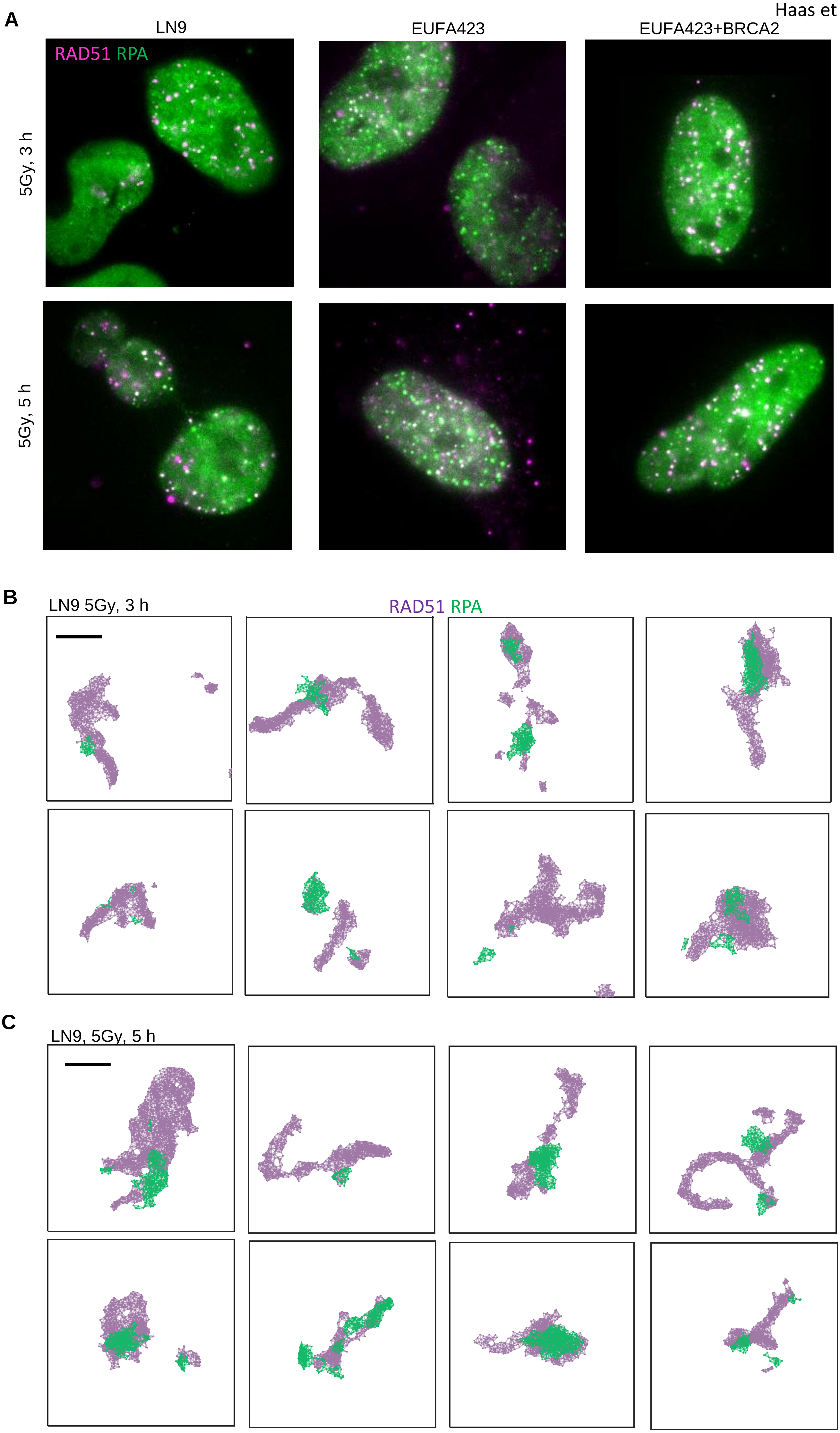
A
LN9
EUFA423
EUFA423+BRCA2
RAD51 RPA
5Gy, 3 h
5Gy, 5 h
B
LN9 5Gy, 3 h
RAD51 RPA
C
LN9, 5Gy, 5 h

Viability after IR (5Gy)

A

EUFA423

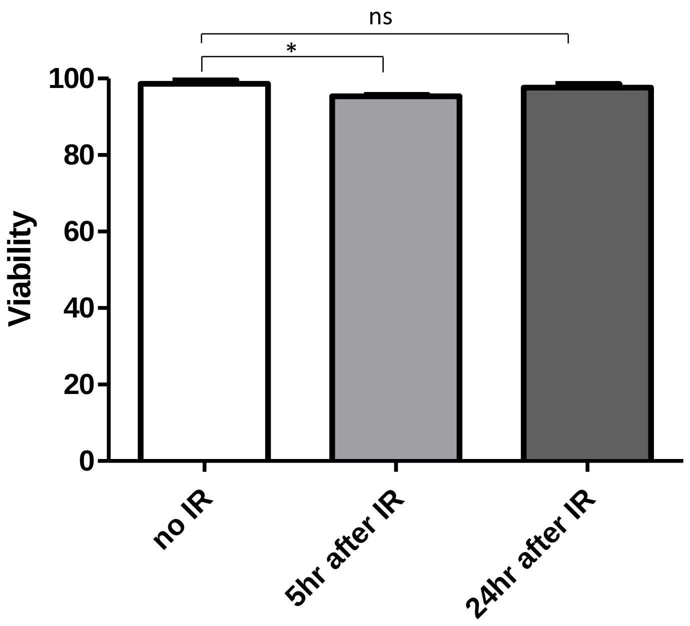

B

EUFA423+BRCA2

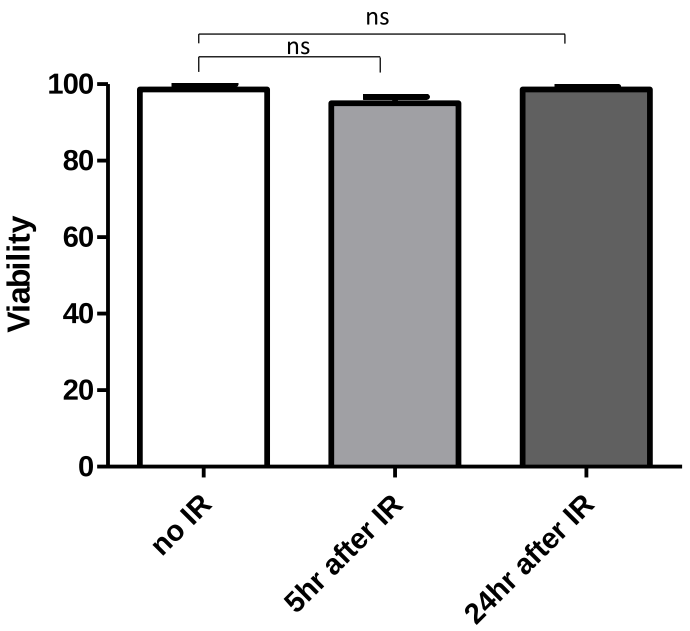

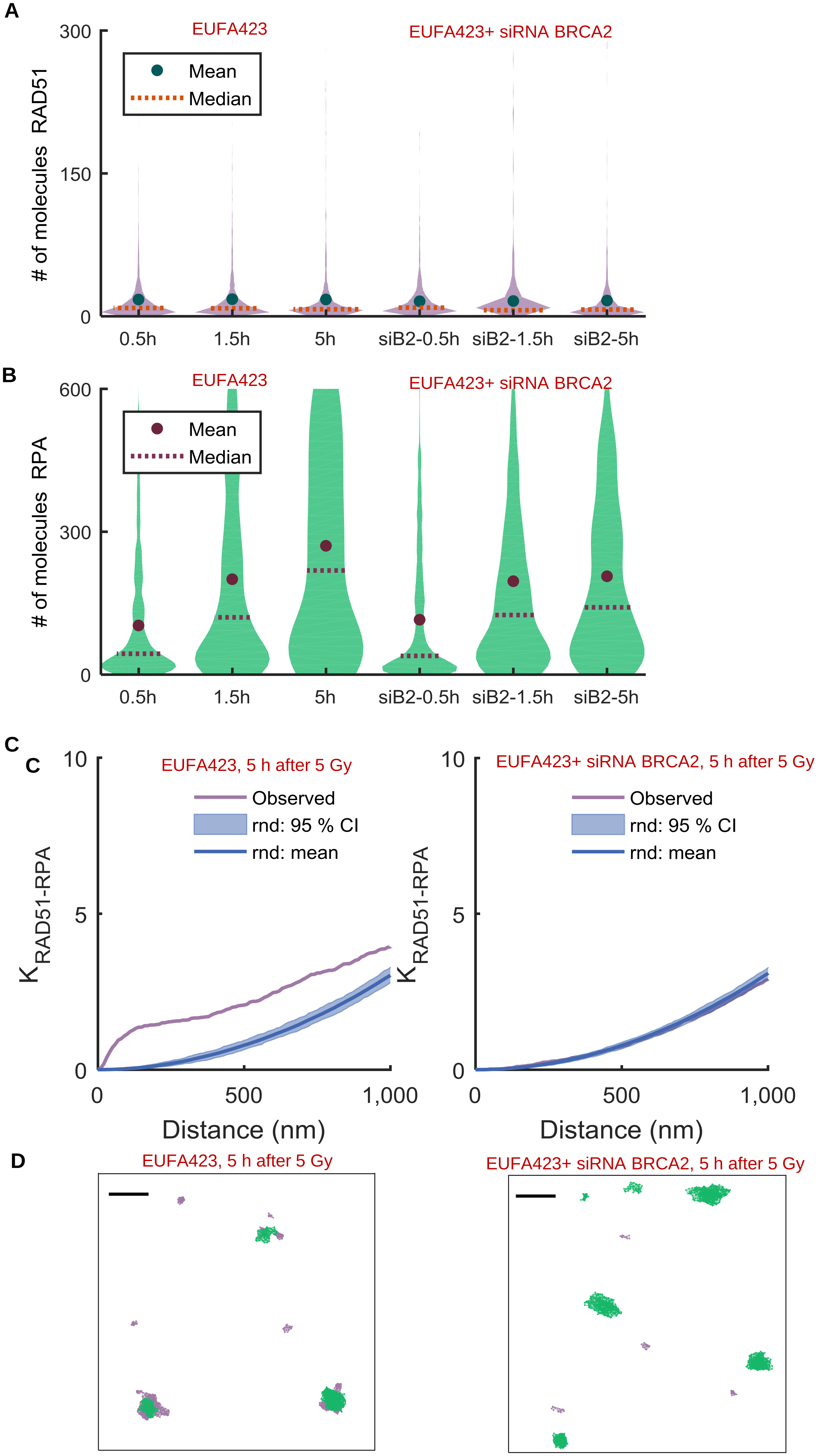
A
EUFA423
EUFA423+ siRNA BRCA2
Mean
Median
# of molecules RAD51
300
150
0
0.5h
1.5h
5h
siB2-0.5h
siB2-1.5h
siB2-5h
B
EUFA423
EUFA423+ siRNA BRCA2
Mean
Median
# of molecules RPA
600
300
0
0.5h
1.5h
5h
siB2-0.5h
siB2-1.5h
siB2-5h
C
C
EUFA423, 5 h after 5 Gy
EUFA423+ siRNA BRCA2, 5 h after 5 Gy
Observed
rnd: 95 % CI
rnd: mean
$K_{RAD51-RPA}$
10
5
0
0
500
1,000
Distance (nm)
D
EUFA423, 5 h after 5 Gy
EUFA423+ siRNA BRCA2, 5 h after 5 Gy
